# SRSF1 and SRSF9 RNA binding proteins promote Wnt signalling-mediated tumorigenesis by enhancing β-catenin biosynthesis

**DOI:** 10.1002/emmm.201202218

**Published:** 2013-04-17

**Authors:** Yu Fu, Binlu Huang, Zhen Shi, Jiayu Han, Ying Wang, Jieqiong Huangfu, Wei Wu

**Affiliations:** 1 Protein Science Laboratory of the Ministry of Education, School of Life Sciences, Tsinghua UniversityBeijing, China

**Keywords:** β-catenin synthesis, oncogene, SRSF1, SRSF9, Wnt signalling

## Abstract

Wnt/β-catenin signalling is widely implicated in embryogenesis, tissue homeostasis and tumorigenesis. The key event in Wnt signalling activation is β-catenin accumulation, which is controlled by both its production and degradation. However, much more emphasis has been placed on the understanding of its degradation. Here, we show that the synthesis of β-catenin protein, which requires a group of serine/arginine-rich splicing factors (SRSF), also contributes to its tumorigenic activity. Overexpression of SRSF1 and SRSF9 promote β-catenin accumulation via the recruitment of β-catenin mRNA and by enhancing its translation in an mTOR-dependent manner. We further demonstrate that, like SRSF1, SRSF9 is also an oncogene, and is frequently overexpressed in multiple types of human tumours. Finally, our results suggest that promoting degradation and blocking production of β-catenin synergistically reduce β-catenin levels under pathological conditions and that a combinational therapy could be a promising approach for the treatment of cancer patients.

## INTRODUCTION

The canonical Wnt/β-catenin signalling pathway plays fundamental roles in regulating cell fate during embryonic development and tissue homeostasis (Chien et al, 2009; Clevers, 2006; Klaus & Birchmeier, 2008; Logan & Nusse, 2004; MacDonald et al, 2009; van Amerongen & Nusse, 2009). It is also crucial for tissue stem cells regulation, and its impairment is heavily linked to tumorigenesis (Clevers & Nusse, 2012; de Sousa et al, 2011; Polakis, 2012b; Schepers & Clevers, 2012). In the canonical Wnt signalling cascade, the protein level of β-catenin, the key effector functioning as a transcriptional coactivator, is critical for its target gene expression and cell fate determination (Jamieson et al, 2012; MacDonald et al, 2009; Valenta et al, 2012). In the absence of Wnt ligand, β-catenin is captured by the scaffold protein, Axin, which facilitates sequential phosphorylation by casein kinase 1 (CK1) and glycogen synthase kinase 3β (GSK3β) in the destruction complex (Liu et al, 2002; Xing et al, 2003). Phosphorylated β-catenin is recognized and ubiquitinated by E3-ubiquitin ligase β-TrCP in an APC- (adenomatous polyposis coli) dependent manner and is subsequently degraded rapidly through the proteasome (Hart et al, 1999; Liu et al, 1999; Rubinfeld et al, 1993; Su et al, 2008). Upon Wnt ligand binding to the Frizzled and LRP5/6 (low decently lipo-protein receptor 5/6) complex, the β-catenin destruction complex becomes dysfunctional by a mechanism that is not fully understood. As a result, the newly synthesized β-catenin accumulates in the cytosol, translocates to the nucleus and forms a complex with transcription factor TCF/LEF, leading to transcriptional activation of the target genes (Archbold et al, 2012; Mosimann et al, 2009). Among these hundreds of target genes, c-myc, cyclin D1 and Axin2 are the most often mentioned ones. β-Catenin accumulation not only occurs upon extracellular Wnt ligand stimulation, but also results intracellularly from mutation or overexpression of other regulatory genes. Genes encoding components of the destruction complex, for example APC or Axin, are often mutated, resulting in β-catenin accumulation in cancer samples (Jin et al, 2003; Morin et al, 1997; Polakis, 2007). Mutations in the β-catenin gene (*CTNNB1*) itself also leads to uncontrolled protein accumulation (Morin et al, 1997). Overexpression of Dvl, ATDC, and deubiquitinating enzyme Fam, which also affects the degradation of β-catenin, has been reported to elevate β-catenin level (Schwarz-Romond et al, 2007; Taya et al, 1999; Wang et al, 2009). β-Catenin protein is believed as a fast turnover protein with a half-life about 1–2 h (Hannoush, 2008; Salic et al, 2000). Theoretically, the final protein level inside a cell should be balanced by its production and degradation together. Therefore, the regulation of its production and degradation should be of equal importance. However, little is known about the regulation of β-catenin protein synthesis, particularly the factors that are involved and are able to enhance this process. HuR, an RNA binding protein, is the only known factor able to stabilize β-catenin mRNA and to enhance β-catenin protein production, and it contributes to β-catenin accumulation in colon cancer (Lopez de Silanes et al, 2003). On the other hand, factors that destabilize β-catenin mRNA or reduce β-catenin protein production have also been identified, including KSRP, eIF6 and Quaking (Bikkavilli & Malbon, 2010; Gherzi et al, 2006; Ji et al, 2008; Ruggiero et al, 2007; Yang et al, 2010). Compared with our understanding of β-catenin degradation, the regulation of β-catenin protein production is far less clear and therefore requires further investigation.

The regulation of protein synthesis occurs at multiple steps including splicing, mRNA transport, formation of the translational initiation complex, and synthesis of the polypeptide. Among many other proteins, the SR proteins (serine/arginine-rich splicing factor, SRSF) are essentially involved in almost every step of the process (Bourgeois et al, 2004; Long & Caceres, 2009; Manley & Tacke, 1996; Shepard & Hertel, 2009; Zhong et al, 2009). The human SR protein family is composed of 12 structurally conserved members (Manley & Krainer, 2010). All SR proteins have one or two N-terminal RNA-binding domains (RBDs; also known as RNA recognition motif [RRM]) that provide RNA-binding specificity, and a C-terminal domain rich in Arg-Ser dipeptides (RS domain) that act as nuclear localization signals and facilitate interaction between different splicing factors (Caceres et al, 1997; Hertel & Graveley, 2005; Manley & Krainer, 2010). Although SR proteins predominantly localize in the nucleus, a subset of them shuttles continuously between the nucleus and the cytoplasm, indicating that SR proteins may function beyond splicing (Sanford et al, 2004; Twyffels et al, 2011). Indeed, considerable findings highlight that SR proteins are involved in many diverse cellular processes in addition to splicing, including mRNA nuclear export, stability control, translation, maintenance of genomic stability and oncogenic transformation (Graveley, 2000; Huang & Steitz, 2005; Twyffels et al, 2011).

The best-studied SR protein is SRSF1, previously called SF2/ASF, which was proved to be a proto-oncogene overexpressed in many cancers (Karni et al, 2007). Several mechanisms were proposed as to how SRSF1 promotes tumour development. Alternative splicing of key mRNAs, including the tumour suppressor BIN1, was proposed to be the major event that caused cell transformation upon SRSF1 overexpression (Karni et al, 2007). SRSF1 is able to activate mTOR pathway which is essential for SRSF1-mediated transformation (Karni et al, 2008; Michlewski et al, 2008). Subsequently, SRSF1 was found to enhance the expression of anti-apoptotic protein Survivin partially via mTOR pathway-dependent mechanism and thus promote tumorigenesis in non-small cell lung cancer (Ezponda et al, 2010). More recently, SRSF1 was demonstrated to cooperate with MYC in promoting mammary epithelial cell transformation (Anczukow et al, 2012). SRSF3 (SRp20) was also suggested to be a proto-oncogene that regulates cell proliferation (Jia et al, 2010). All these reports suggest that overexpressed SR proteins contribute to cancer progression via multiple mechanisms.

In this study, we demonstrate that SRSF1 and SRSF9, two closely related SR proteins, are able to promote Wnt signalling by elevating β-catenin levels. Biochemical experiments indicate that both SR proteins bind to β-catenin mRNA and enhance β-catenin synthesis. Importantly, the β-catenin accumulation induced by either Wnt ligand stimulation or mutation of APC/β-catenin in colon cancer cells is dependent on SRSF1 and SRSF9. Furthermore, we provide evidence suggesting that SRSF9, like SRSF1, is also a proto-oncogene and β-catenin is involved in SRSF1- and SRSF9-induced cell transformation. These results not only suggest β-catenin is a novel target of cancerous SR proteins, but also highlight the significant contribution of protein synthesis in β-catenin accumulation.

## RESULTS

### A subset of SR proteins enhances β-catenin accumulation and Wnt signalling activation

**Figure 1:**
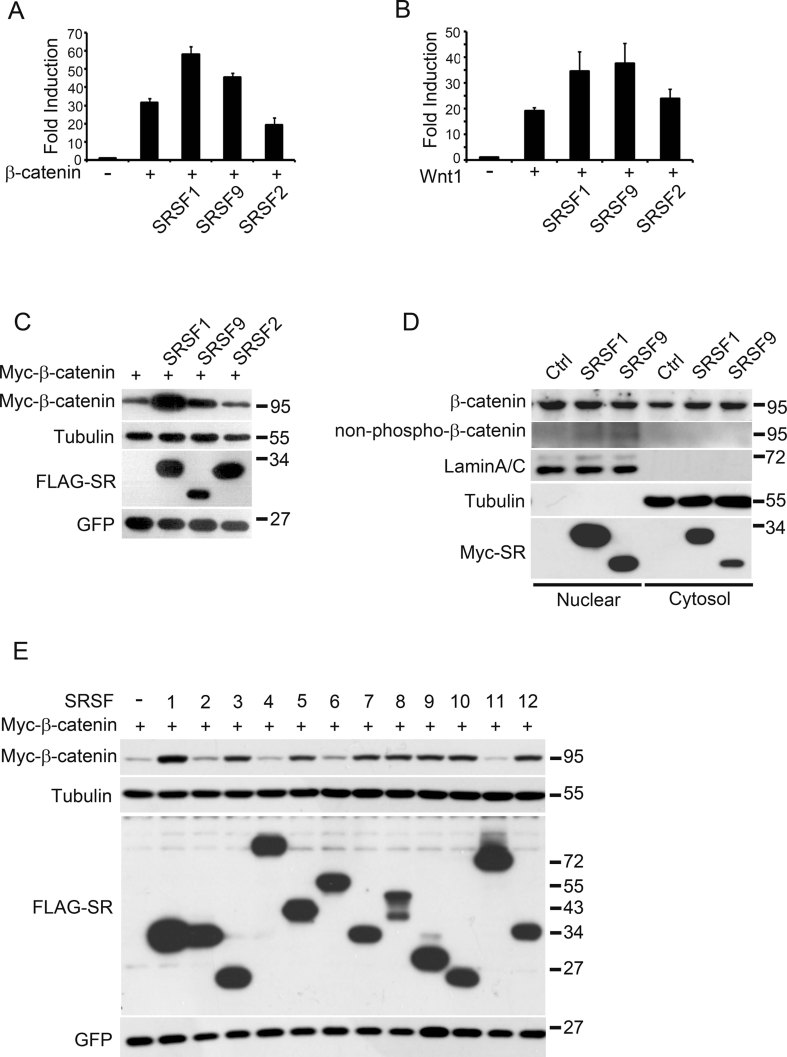
A subset of SR proteins promotes β-catenin accumulation and Wnt signalling activation. A,B. SRSF1 and SRSF9, but not SRSF2 enhanced β-catenin-(A) or Wnt1-(B) activated reporter expression. HEK293T cells were transfected with Wnt-responsive TOPFLASH luciferase reporter together with indicated plasmids. C. SRSF1 and SRSF9, but not SRSF2 enhanced β-catenin accumulation. Myc-β-catenin/GFP mix was transfected alone or co-transfected with FLAG-tagged SRs as indicated into HEK293T cells and total cell lysate was separated by SDS–PAGE and proceeded by Western blotting using different antibodies as indicated. Equal GFP plasmid was co-transfected with each sample to control transfection efficiency. Tubulin was used to control equal loading. D. Endogenous β-catenin protein level was also elevated by SRSF1 or SRSF9 over-expression in transfected HEK293T cells. Control, FLAG-SRSF1 or FLAG-SRSF9 transfected HEK293T cells were fractionated into the cytosol and nuclear parts and proceeded by SDS–PAGE and Western blotting with indicated antibodies. E. SRSF1, 3, 5, 7, 8, 9, 10, 12, but not SRSF2, 4, 6, 11 promote β-catenin accumulation in HEK293T cells. Myc-β-catenin/GFP mix was co-transfected with indicated SRSF plasmids and total cell lysates were proceeded by SDS–PAGE and Western blotting.

In a Wnt-responsive reporter-based functional screen carried out in HEK293T cells (Davidson et al, 2005), SRSF9 (serine/arginine-rich splicing factor 9), the *Xenopus tropicalis* homologue of human SRSF9, was identified as potent Wnt signalling enhancer (Supporting Information Fig S1A). Since SRSF9 is closely related to SRSF1, which is studied extensively, we focused our characterization on these two proteins in the following study. Using Wnt-responsive reporter assay, we found that human SRSF1 and SRSF9 were also able to enhance Wnt1- as well as β-catenin-induced reporter expression, whereas SRSF2 could not (Fig 1A and B). Their enhancing activity was Wnt/β-catenin signalling-specific since neither TGF-β nor Notch signalling was significantly affected (Supporting Information Fig S1B and C).

Since β-catenin accumulation is the key event in Wnt signalling activation, we measured β-catenin protein level after SRSF1 or SRSF9 co-expression. As indicated in Fig 1C, total β-catenin level was significantly elevated upon SRSF1 or SRSF9 co-transfection, but not with SRSF2, consistent with the reporter assay results (Fig 1A). These β-catenin proteins were expressed from transfected plasmids (constructed in pCS2+ vector), in which the β-catenin coding region was flanked by alpha-globin 5′UTR and SV40 3′UTR/polyadenylation signal. To exclude the possibility that these artificial UTRs might contribute to β-catenin protein production, we subcloned full-length human β-catenin cDNA (accession number NM_001904.3) into a vector without exogenous UTR (pEGFP-C1 vector digested by *Nhe*I and *Mlu*I). Co-transfection of SRSF1 or SRSF9 with this ‘real’ human β-catenin cDNA also produced much more β-catenin protein (Supporting Information Fig S1D). Finally, to test whether SRSF1 and SRSF9 are able to elevate endogenous β-catenin levels, we measured β-catenin protein in the cytosolic and nuclear fractions of transfected HEK293T cells. As shown in Fig 1D, β-catenin levels in the cytosol fraction were clearly elevated upon SRSF1 or SRSF9 over-expression. In the nucleus, however, the total β-catenin protein level was not significantly elevated but the active, non-phosphorylated form of β-catenin (non-phospho-β-catenin) was slightly but constantly enhanced. The effect on β-catenin protein was rather specific since several other Wnt signal pathway components, including Dvl2, LRP6 and GSK3β, were not changed (Supporting Information Fig S1E and F). These results suggested that SRSF1 and SRSF9, but not SRSF2, were able to potentiate Wnt/β-catenin signalling via elevation of β-catenin protein level.

The canonical human SR protein family contains 12 members (Manley & Krainer, 2010). To test whether other SR proteins have the same effect on β-catenin accumulation, we cloned the cDNAs of all 12 human SR genes. In a co-transfection assay, SRSF1, 3, 5, 7, 8, 9, 10 and 12 were all able to cause β-catenin accumulation, while SRSF2, 4, 6 and 11 could not (Fig 1E). These results suggest that a subset of human SR proteins was able to promote β-catenin accumulation and hence potentiate Wnt signalling. Moreover, these results also suggest that the SR family proteins could be classified into two distinct groups according to their ability to elevate β-catenin.

### SRSF1 and SRSF9 enhance β-catenin synthesis in an mTOR-dependent manner

**Figure 2:**
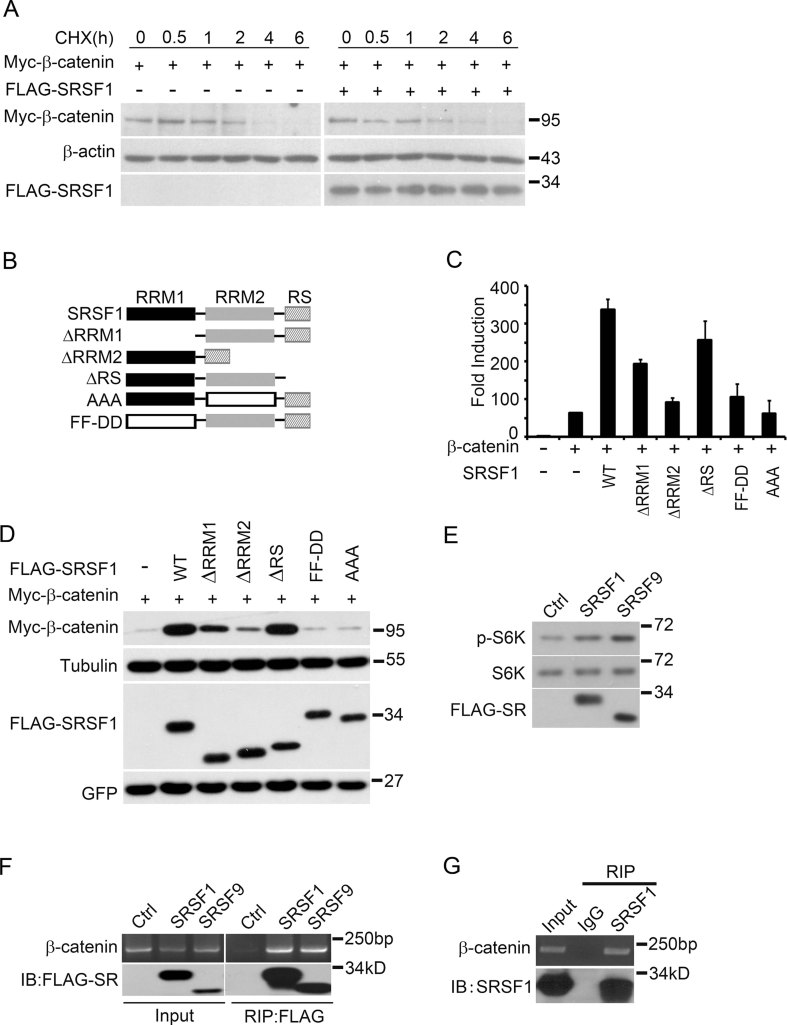
Mechanism of β-catenin accumulation induced by SRSF1. A. β-Catenin protein stability was not affected by over-expressed SRSF1. Myc-β-catenin was transfected alone or co-transfected with FLAG-SRSF1 and 36 h later cells were treated with cycloheximide (CHX, 100 µg/ml) and then harvested at the indicated time points (hours post adding). In order to have the same β-catenin level at the starting point, 300 ng β-catenin plasmid was used for tranfection alone and 200 ng for co-transfection with 10 ng SRSF1 plasmid. B. Schematic diagram showing mutations and deletions of SRSF1. C. Wnt-responsive reporter assay showing that SRSF1 and its deletions/mutations exerted different activities on β-catenin signalling. Results are representative of at least three experiments. D. SRSF1 and its deletions/mutations exerted different activities on β-catenin protein production. Myc-β-catenin/GFP mix was transfected alone or co-transfected into HEK293T cells with indicated SRSF1 mutations/deletions and the total cell lysates were proceeded by SDS–PAGE and Western blotting. WT means wide type. E. S6K phosphorylation (an indication of mTOR activation) was enhanced by over-expression of SRSF1 or SRSF9. HEK293T cells were transfected as indicated and 36 h later, total cell lysates were prepared and analysed by SDS–PAGE and Western blotting with indicated antibodies. p-S6K means antibody recognizes phosphorylated S6K. F. RIP results showing that over-expressed FLAG-SRSF1 and FLAG-SRSF9 bound to endogenous β-catenin mRNA. HEK293T cells were transfected as indicated and 36 h later, cell lysates were prepared and SR proteins were immunoprecipitated using FLAG-beads and bound RNA was extracted and analysed using RT-PCR. Upper row shows RT-PCR results and bottom row shows Western blotting results. G. RIP results showing that endogenous SRSF1 bound to endogenous β-catenin mRNA. Total lysates from HEK293T cells were immunoprecipitated with anti-SRSF1 antibody or a control IgG immunoglobulin (IgG; bottom row) and bound RNA was extracted and analysed by RT-PCR to detect endogenous β-catenin mRNA (upper row).

Cellular β-catenin level at steady state is determined by its production and degradation rate. Our results show that the half-life of β-catenin protein did not change upon overexpression of SRSF1 or SRSF9 (Fig 2A, Supporting Information Fig S2A), suggesting that SR protein-induced β-catenin accumulation was not due to decreased β-catenin degradation. To further validate this hypothesis, we show that the level of S37A-β-catenin (SA-β-catenin), a stabilized form in which the GSK3 phosphorylation site is mutated, could be further elevated by overexpression of SRSF1 or SRSF9 (Supporting Information Fig S2C), suggesting that the effect of SR proteins was independent of β-catenin degradation. Furthermore, the β-catenin mRNA levels did not change upon overexpression of SR proteins (Supporting Information Fig S2B). Therefore, it is most likely that SR proteins regulate β-catenin at the mRNA translational step.

Next, we assayed the domain contribution of SR proteins in mediating β-catenin accumulation. Structurally SRSF1 and SRSF9 are quite similar, containing RRM1, RMM2 and RS domains (Screaton et al, 1995). RRM1 and RRM2 are responsible for RNA binding, and the RS domain is required for their nuclear localization and splicing activity (Philipps et al, 2003; Shepard & Hertel, 2009). Full-length SR proteins are largely localized to the nucleus, with a small fraction shuttling between cytosol and nuclei, whereas RS domain-deleted SR proteins are distributed more in the cytosol (Caceres et al, 1997). SRSF1 or SRSF9 without its RS domain (ΔRS) retained Wnt signalling and β-catenin promoting activity (Fig 2B–D and Supporting Information Fig S2D–F), suggesting the nuclear localization or splicing activity was not stringently required. This is consistent with the fact that β-catenin production from transfected plasmids is splicing-independent (which contains no intron). These results suggested that SRSF1 and SRSF9 likely promoted β-catenin accumulation in the cytosol.

By contrast, both RRM1 and RRM2 were required for SR-promoted β-catenin accumulation and Wnt signalling (Fig 2B–D and Supporting Information Fig S2D–F). It has been reported that a FF–DD mutation within the RRM1 domain impaired RNA binding of SRSF1. We therefore tested corresponding mutants of SRSF1 and SRSF9 and found indeed, they had lost β-catenin promoting activity, suggesting the RNA binding was necessary. In addition to splicing, SRSF1 has been suggested to regulate protein translation by recruiting mTOR via its RRM2 domain, and an AAA mutation within this domain abolishes this interaction (Michlewski et al, 2008). We also created the AAA mutation in SRSF1 and SRSF9 and revealed that these mutations were completely non-functional in promoting β-catenin accumulation (Fig 2B–D and Supporting Information Fig S2D–F). These results suggested that RNA binding and mTOR interacting motifs were necessary but splicing activity was dispensable for SRSF1 and SRSF9 to promote β-catenin accumulation.

SRSF1 has been reported to have a role in promoting mTOR-mediated protein translation (Karni et al, 2008; Michlewski et al, 2008), yet that for SRSF9 has not been verified. We performed Western blotting experiments after overexpression of SRSF1 and SRSF9 and observed enhanced p70S6K phosphorylation, an indication of mTOR activation (Fig 2E). This result suggested that, like SRSF1, SRSF9 was also able to promote mTOR activation upon overexpression. In order to confirm that SRSF1 and SRSF9 were indeed promoting β-catenin protein synthesis, we carried out RNA immunoprecipitation (RIP) experiments. Both overexpressed FLAG-tagged-SRSF1 and -SRSF9 (Fig 2F), as well as endogenous SRSF1 (Fig 2G), were able to pull-down β-catenin mRNA specifically. As a final proof, we performed sucrose sedimentation experiments and observed increased β-catenin mRNA levels in ribosome fractions upon SRSF1 overexpression (Supporting Information Fig S2G). Collectively, these results strongly suggest that SRSF1 and SRSF9 promote β-catenin accumulation via binding β-catenin mRNA and enhancing its protein synthesis in an mTOR-dependent manner. The splicing activity of SR proteins is rather not related to this process.

### SRSF1 and SRSF9 are required for β-catenin accumulation and Wnt signalling

**Figure 3:**
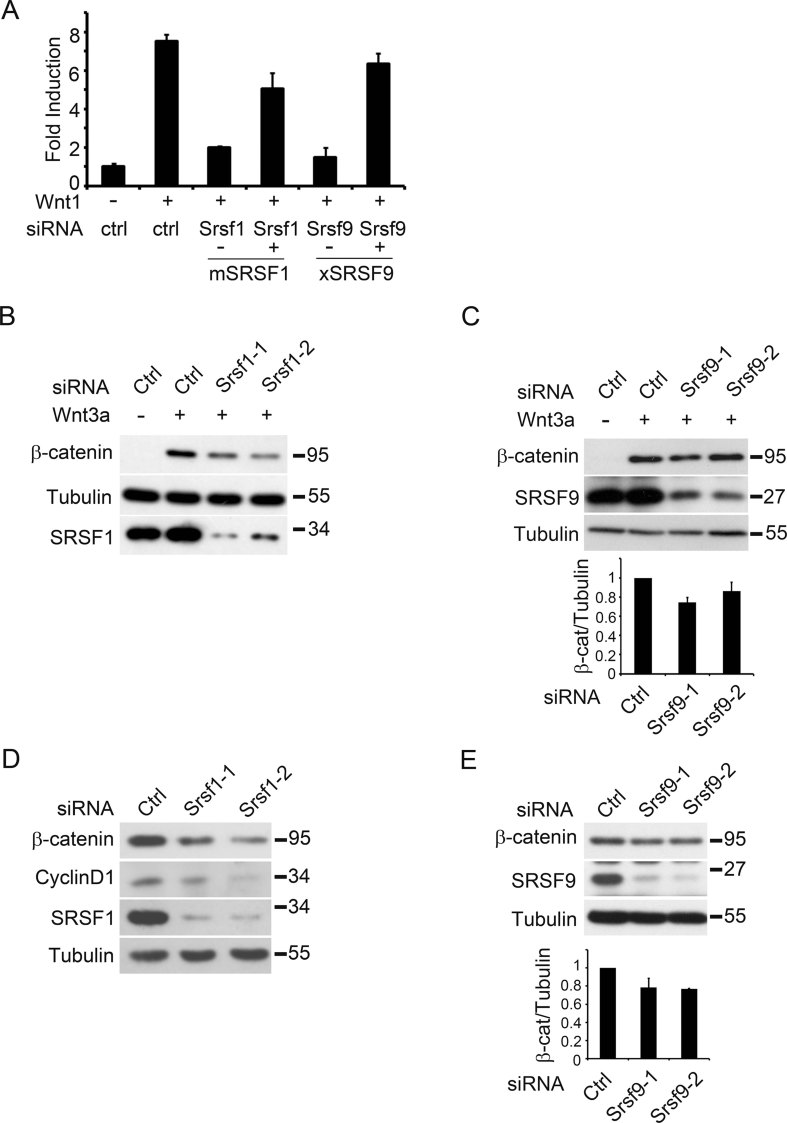
SRSF1 and SRSF9 are required for Wnt-induced β-catenin accumulation. A. SRSF1 and SRSF9 are required for Wnt1-stimulated reporter expression. HEK293T cells were transfected with Wnt-responsive TOPFLASH reporter, Wnt1 plasmids, human SRSF1-siRNA-2 and SRSF9-siRNA-1 as indicated. Mouse SRSF1 (mSRSF1) or *Xenopus* SRSF9 (xSRSF9) was able to rescue the β-catenin reporter activation. B,C. RKO cells were transfected with siRNAs as indicated and 48 h later, the cells were treated with Wnt3a protein (100 ng/ml) for 1 h and total cell lysates were harvested and proceeded by SDS–PAGE and Western blotting. The relative expression of β-catenin against Tubulin were quantified with three independent experiments. D,E. HCT116 cells were transfected with siRNAs as indicated and 48 h later, total cell lysates were prepared and proceeded by SDS–PAGE and Western blotting with indicated antibodies. The relative expression of β-catenin against Tubulin were quantified with three independent experiments.

Cellular β-catenin proteins that transduce Wnt signalling are under the control of phosphorylation/ubiquitination-mediated proteasome degradation, therefore, Wnt signalling-induced β-catenin accumulation depends extensively on newly synthesized proteins (Clevers & Nusse, 2012). To determine whether SRSF1 and SRSF9 are required for Wnt signalling, HEK293T cells were transfected with siRNA against SRSF1 or SRSF9 and Wnt-responsive reporter assays were performed. As shown in Fig 3A, both siSRSF1 and siSRSF9 reduced Wnt signalling. The siRNA effects were specific since the down-regulation of Wnt signalling was rescued by the introduction of mouse SRSF1 or *Xenopus* SRSF9, respectively, which were resistant to corresponding siRNA. To directly demonstrate that SR proteins participated in β-catenin synthesis, we knocked-down SRSF1 or SRSF9 in RKO cells, a human colon cancer cell line in which Wnt signalling is relatively low. Wnt3a treatment induced rapid and dramatic β-catenin accumulation, and the induction was reduced in SRSF1 or SRSF9 knockdown cells (Fig 3B and C). These results suggested that SRSF1 and SRSF9 were indeed involved in Wnt signalling-induced β-catenin accumulation. Next, we asked whether β-catenin accumulation in colon cancer cell lines harbouring mutations impaired β-catenin degradation, for example HCT116 with β-catenin mutation or SW480/SW620 cells with APC mutation, was also dependent on SR proteins. Knockdown of SRSF1 or SRSF9 in these cell lines also reduced β-catenin levels (Figs 3D, E and 6C, D). Importantly, Cyclin D1, one of the major Wnt target genes, was also down-regulated (Fig 3D). From these results, we concluded that SRSF1 and SRSF9 are required not only for Wnt-induced but also tumorigenic β-catenin accumulation in cancer cells harbouring mutations that impair β-catenin degradation.

### SRSF9 is a proto-oncogene

**Figure 4:**
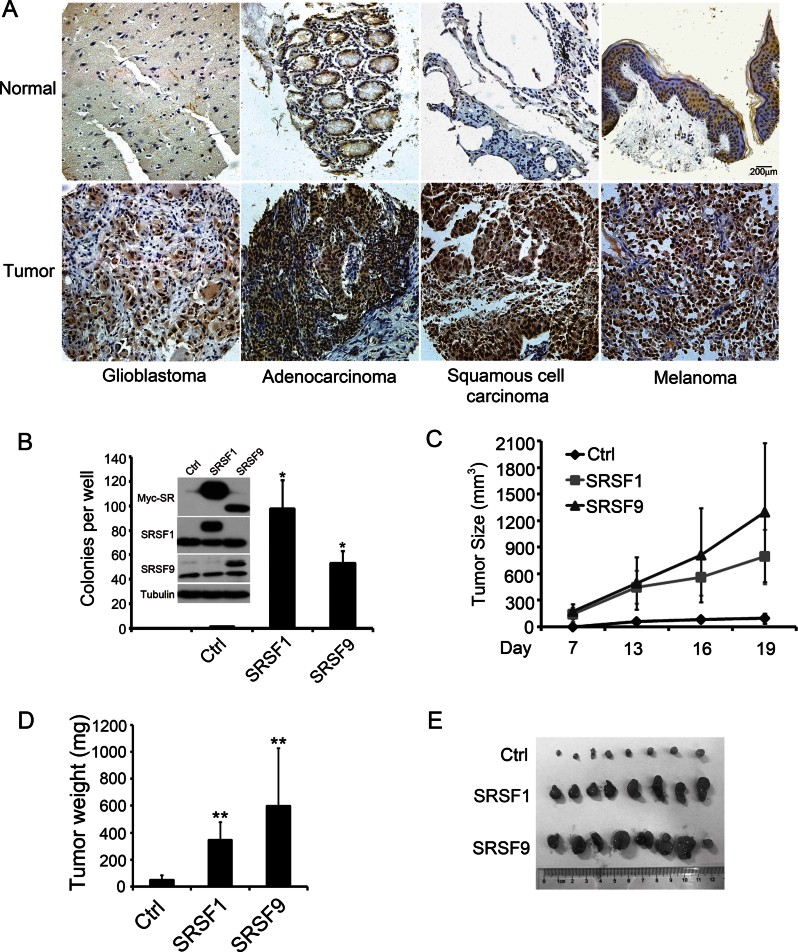
SRSF9 is a proto-oncogene. A. Representative immunohistochemical images of SRSF9 expression in human cancer samples (lower panels) and paracancerous normal tissue (upper panels). Scale bar, 200 µm. B. Quantification of colony formation assay results with control NIH3T3 cells or cells stably expressing SRSF1 or SRSF9. The expression levels were shown in inset. *p* Values in pairwise comparisons to the control group, *p < 0.05. (mean ± SD, *n* = 3) C–E. Tumour volumes were evaluated in mice injected with 2.5 × 10^5^ control NIH3T3 cells or cells stably expressing SRSF1 or SRSF9 at the indicated days post injection. Each group contained eight mice. Error bars, s.d. The tumour weight (D) was measured when the mice were sacrificed on Day 19 (mean ± SD, *n* = 8, **p < 0.01). Tumours from each group are shown (E).

Because over-accumulated β-catenin is tumorigenic, and SRSF1 has been suggested as a proto-oncogene (Anczukow et al, 2012; Ezponda et al, 2010; Karni et al, 2007), we hypothesized that SRSF9 may also be an oncogenic SR protein. To verify whether SRSF9 is overexpressed in cancer samples, we performed immunohistochemistry staining on cancer arrays with different tissue origins (Fig 4A). The results indicated that SRSF9 expression levels, in comparison with corresponding normal tissue, were elevated with high frequency in multiple types of cancer samples including glioblastoma (18/20), colon adenocarcinoma (18/20), squamous cell lung carcinoma (19/20) and malignant melanoma (15/20). To further confirm that SRSF9 gene is indeed over-expressed in tumour samples, we searched the oncomine cancer microarray database (http://www.oncomine.com), and found a significant increase (*p* < 0.05) of SRSF9 (SRp30c) expression in tumour tissues over the expression in corresponding normal tissues in 156 of 360 studies. Fisher's meta analysis indicated that the observed increase in those paired studies was extremely significant (meta *p*-value < 0.0001). These results demonstrate that SRSF9 is broadly upregulated in tumour samples.

To test the oncogenic activity of SRSF9, we applied the standard NIH3T3 cell transformation assay. Control, SRSF1- or SRSF9-overexpressing cell lines were established, and their tumorigenic potential were verified in anchorage-dependent colony formation assays and nude mice xenograft experiments. Consistent with previous reports (Anczukow et al, 2012; Karni et al, 2007), SRSF1 overexpression promoted colony formation. Similar results were obtained in SRSF9-overexpressing NIH3T3 cells (Fig 4B). Furthermore, we implanted these cells in nude mice and measured tumour growth throughout the subsequent 3 weeks. As shown in Fig 4C–E, both SRSF1- and SRSF9-overexpressing cells were tumorigenic and the SRSF9-induced tumours seemed even larger than those of SRSF1. Together, these data suggested that SRSF9 is a proto-oncogene.

### β-Catenin is partially required for SR protein-mediated cell transformation

**Figure 5:**
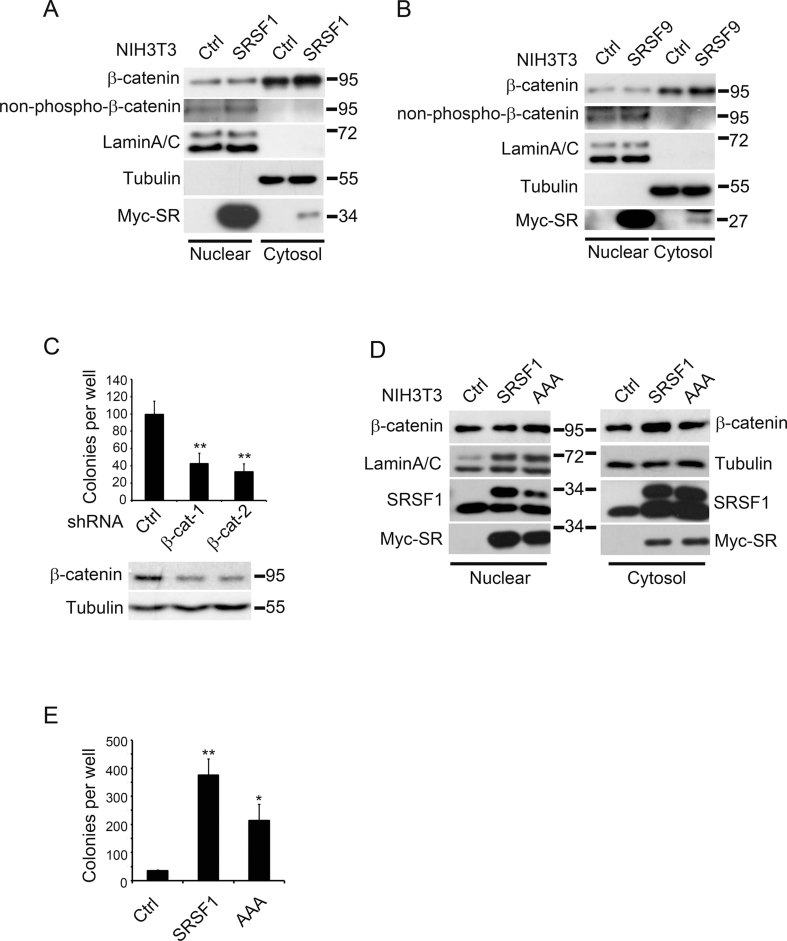
β-Catenin is partially required for SR proteins-mediated cell transformation. A,B. Control NIH3T3 cells, cells stably expressing myc-SRSF1 (A) or myc-SRSF9 (B) were fractionated into cytosol and nuclear parts and the lysates were proceeded for SDS–PAGE and Western blotting with indicated antibodies. Endogenous β-catenin protein level in the cytosolic fraction was significantly enhanced by over-expression of either SR protein. Active β-catenin (non-phospho-β-catenin) was increased in the nuclear fraction by SR over-expression. C. Down-regulation of β-catenin in SRSF1-over-expressing NIH3T3 cells reduced their capacity of colony formation (mean ± SD, *n* = 3, **p < 0.01). Two independent shRNA constructs were applied to knock-down β-catenin and their efficiency was shown below by Western blotting. D,E. AAA-SRSF1 was not as active as wide type SRSF1 in causing β-catenin accumulation and cell transformation. (D) Cell fractionation experiment was performed with control NIH3T3 cells, cells stably expressing SRSF1 or AAA-SRSF1. Please note that the wide type SRSF1 caused cytosolic β-catenin elevation while AAA-SRSF1 did not. (E) Colony formation assay with the same cells as in (D). *p* Values in pairwise comparisons to the control: **p = 0.009, *p = 0.032.

The above results suggested that β-catenin could be another major target of SRSF1 as well as SRSF9, in addition to previously identified BIN1 and Survivin (Ezponda et al, 2010; Karni et al, 2007). In order to verify the contribution of β-catenin to SR-induced cell transformation, we first detected β-catenin accumulation in SRSF1- or SRSF9-overexpressing NIH3T3 cells. Similar to the results shown in Fig 1D, β-catenin levels in the cytosol were clearly elevated upon SRSF1 or SRSF9 overexpression, while in the nucleus, although the total β-catenin was not significantly elevated, the active form was constantly enhanced (Fig 5A and B). More importantly, when we knocked-down β-catenin expression in SRSF1-transformed NIH3T3 cells, their colony formation ability was dramatically reduced (Fig 5C), suggesting that β-catenin was indeed required for SRSF1-mediated cell transformation. To further confirm that the translation-promoting ability of SRSF1 was involved in β-catenin accumulation and cell transformation, we established NIH3T3 cells overexpressing AAA-SRSF1, which have normal splicing activity but impaired mTOR interaction and therefore reduced translation-promoting activity. As shown in Fig 5D, elevation of cytosolic β-catenin did not occur in AAA-SRSF1-expressing cells, consistent with the result shown in Fig 2D. As expected, colony formation of AAA-SRSF1-expressing cells was reduced (Fig 5E). These results suggested that β-catenin was one of the major targets of both SRSF1 and SRSF9 in promoting cell transformation.

Together, these results indicated that in NIH3T3 cells transformed by SRSF1 or SRSF9 over-expression, not only that β-catenin level is elevated, but also that β-catenin is partially required for this cell transformation.

### SRSF1 and SRSF9 are involved in cancerous cell proliferation

**Figure 6:**
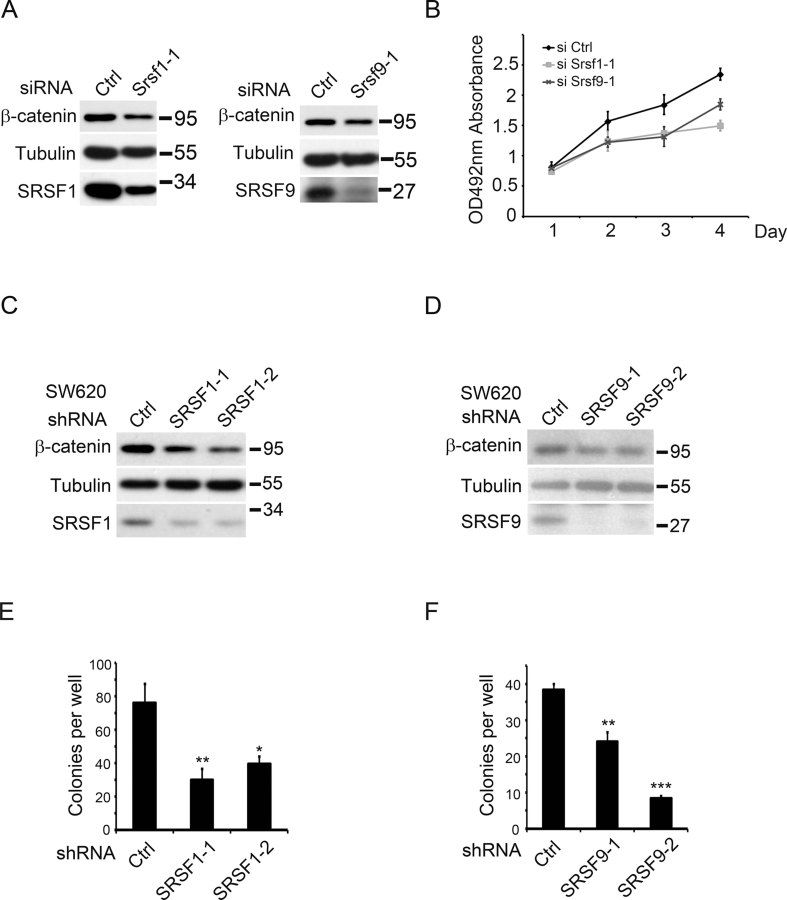
SRSF1 and SRSF9 are required for colon cancer cell proliferation. A. Western blotting results showing that knock-down SRSF1 or SRSF9 in HCT116 cells by indicated siRNA down-regulated β-catenin protein level. B. MTS cell proliferation assay results showing that knock-down of SRSF1 or SRSF9 in HCT116 cells by indicated siRNA reduced cell proliferation. Results represent at least three independent biological replicates. Error bars, s.d. C,D. Western blotting results showing that knock-down SRSF1 (C) or SRSF9 (D) in SW620 cells by indicated shRNA reduced β-catenin protein level. E,F. Colony formation assay results showing that knock-down of SRSF1 (E) or SRSF9 (F) in SW620 cells by indicated shRNA reduced cell proliferation and tumorigenic activity. Results represent three independent biological replicates. *p* Values in pairwise comparisons to the control: *p < 0.05, **p < 0.01, ***p < 0.001.

The results from the above-mentioned immunohistochemistry experiments, as well as previous reports, indicated that SRSF1 and SRSF9 were frequently overexpressed in several types of tumour samples; thus, we next addressed their contribution in cancer cell proliferation. We monitored their expression in colon cancer cell lines including SW480, SW620, HCT116, HT29, RKO, and detected relatively high expression in HCT116 and SW620 cells (Supporting Information Fig S3). We then applied siRNAs against SRSF1 and SRSF9 to HCT116 cells and measured their proliferation. As shown in Fig 6A and B, β-catenin protein levels were reduced upon SR down-regulation, and consistently, cell proliferation was also reduced. We then reduced SRSF1 and SRSF9 expression separately in SW620 cells using a shRNA-mediated gene knockdown approach, and detected β-catenin levels as well as the colony formation ability. As shown in Fig 6C and D, β-catenin protein levels were down-regulated in SRSF1 or SRSF9 knockdown cells. Consistently, colony formation capacity in soft agar was also reduced in these cells (Fig 6E and F). Taken together, these results suggest that overexpressed SRSF1 and SRSF9, at least partially, contribute to cancerous cell proliferation via regulating β-catenin level.

### Reducing cancerous β-catenin level by simultaneously enhancement of degradation and blockade of synthesis

**Figure 7:**
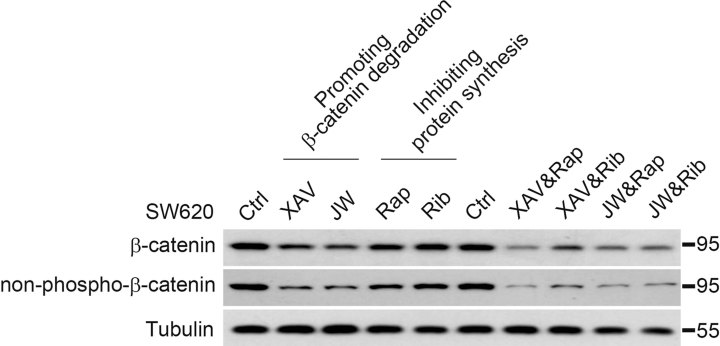
A combined approach to decrease β-catenin level in colon cancer cells. SW620 cells were treated with indicated small molecules alone or in combination for 24 h and the total cell lysates were proceeded for SDS–PAGE and Western blotting with antibodies against total or non-phospho-β-catenin. Small molecules used were XAV939 (XAV, 2 µM), JW74 (JW, 2 µM), Rapamycin (Rap, 200 nM), Ribavirin (Rib, 10 µM).

Reducing β-catenin level is considered as a very promising approach to target cancerous cell proliferation and thus cancer therapy. Luckily, several small molecules were recently identified to promote β-catenin degradation via diverse mechanisms, which provided candidate leading compounds for drug development (Polakis, 2012a). Furthermore, inhibition of mTOR or eukaryotic translation initiation factors (eIFs) has also been applied for cancer treatment (Meric & Hunt, 2002; Silvera et al, 2010). Our results suggested that the accumulation of β-catenin in cancer samples resulted from both impaired degradation as well as enhanced protein synthesis; therefore, simultaneous reversal of these two independent branches should be more effective than manipulation of either of them alone. As a proof of principle experiment, we took XAV939 and JW74 as compounds that promote β-catenin degradation via blockade of tankyrase and therefore stabilizing Axin (Huang et al, 2009; Waaler et al, 2011). Rapamycin, an mTOR inhibitor and Ribavirin, an eIF4E inhibitor, were applied to block protein synthesis (Ballou & Lin, 2008; Kentsis et al, 2004). After careful titration, cooperative effects in reducing β-catenin level in SW620 cells were observed between the degradation enhancers and the translation inhibitors, with XAV939/rapamycin combined having the most significant effect (Fig 7). Importantly, the transcriptional active, non-phospho-β-catenin was also significantly reduced. These results demonstrated, in principle, that these two approaches could be simultaneously applied for cancer therapy against Wnt signalling over-activated tumours.

## DISCUSSION

Our results demonstrated that SRSF1 and SRSF9 are able to promote β-catenin protein synthesis. We further showed that these SR proteins are generally involved in Wnt- or upstream mutation-induced β-catenin accumulation. β-catenin therefore is proposed as a target of SR proteins in tumorigenesis upon their overexpression. Regarding β-catenin over-accumulation in cancer cells, our study emphasizes that the impairment of degradation and enhancement of synthesis make simultaneous contributions and therefore, approaches targeting both paths should be considered during therapy.

### SRSF9 is a proto-oncogene

Among the 12 canonical SR splicing factors, SRSF1 and SRSF3 have been suggested as proto-oncogenes (Anczukow et al, 2012; Ezponda et al, 2010; Jia et al, 2010; Karni et al, 2007). SRSF2 caused NIH3T3 cell transformation in colony formation assays but failed in xenograft assays. In the same report, SRSF6 was indicated inactive in both assays (Karni et al, 2007). More recently, SRSF9 was implicated in the proliferation of a bladder cancer cell line via an unknown mechanism (Yoshino et al, 2012). Here, we provided compelling evidence to demonstrate that SRSF9 is a proto-oncogene: (i) SRSF9 is frequently overexpressed in a wide range of tumour types; (ii) SRSF9 overexpression is able to transform NIH3T3 cells to form colonies *in vitro* and to form tumours in nude mice; (iii) down-regulation of SRSF9 in colon cancer cell lines reduced their colony formation ability. With the exception of the above-mentioned SR proteins, the other members of this protein family have not been studied regarding their role in tumorigenesis. Our results suggested that β-catenin could be a target of some SR proteins, and their ability to cause β-catenin accumulation might be an indication of the tumorigenic activity. This is consistent with the correlation that all known oncogenic SR proteins (SRSF1, SRSF3 and SRSF9) are able to promote β-catenin accumulation, and all non-tumorigenic ones (SRSF2 and SRSF6) cannot. These results promote us to speculate that there are additional canonical SR proteins that are potentially oncogenic. It would be interesting in the future to systemically verify, among all canonical SR proteins, their expression in tumour samples as well as their tumorigenic activities, assuming specific antibodies are available.

### β-Catenin is a novel target of a subset of SR proteins

How a splicing factor promotes tumorigenesis is not obvious at first glance. The physiological function of SR proteins is implicated in genomic stability, pre-mRNA splicing, RNA transportation, RNA stability control and translation. SRSF1 was the first SR protein shown to be oncogenic via promoting alternative splicing of oncogenes including BIN1, MNK2 and S6K1 (Karni et al, 2007). More recently, TEAD1, BIM and Caspase-9 were identified as oncogenic splicing targets (Anczukow et al, 2012; Karni et al, 2007; Shultz et al, 2011). In 2010, the anti-apoptotic protein Survivin was identified as the first translational oncogenic target of SRSF1 in non-small cell lung cancer. SRSF1 was able to promote Survivin protein translation in an mTOR-dependent manner, and was correlated with Survivin expression in tumour samples (Ezponda et al, 2010). Here, we identified SRSF1 and SRSF9 as enhancers of Wnt/β-catenin signalling in an unbiased expression screen. We further provided evidence to suggest that the oncogene β-catenin, the key effector of the Wnt/β-catenin signalling pathway, is a translational target of SRSF1 and SRSF9. Our results indicated that: (i) SR proteins bind to β-catenin mRNA and enhance its protein production without interfering with its degradation; (ii) SR proteins' splicing activity is not absolutely required, but their mRNA binding and mTOR activation domains are indispensable for promoting β-catenin accumulation; (iii) these SR proteins are required for β-catenin accumulation; and (iv) β-catenin is critically involved in SRSF1- and SRSF9-induced cell transformation and tumorigenesis. Our results also suggested that the 12 canonical SR proteins have distinct ability to act on β-catenin, supporting the idea that this effect is specific. Our conclusion is strongly supported by previous reports showing that the tumorigenic activity of SRSF1 is mTOR- and therefore translation-promoting activity-dependent (Anczukow et al, 2012; Karni et al, 2008). Results from this study and previous investigations support the idea that SR proteins may contribute to tumorigenesis by affecting several aspects of the cellular processes, including alternative splicing and the translational state of critical oncogenes or tumour suppresser genes. Considering that each SR protein may recognize hundreds of different mRNAs (Long & Caceres, 2009), it is reasonable to speculate that overexpressed SR protein may cause global changes in cells. A recent report suggested that SRSF1, as a transcriptional target of MYC, played an important role mediating the oncogenic activity of MYC (Das et al, 2012). All these studies gradually placed SR proteins in a central position during cell transformation, as they are in physiological conditions, and suggested that targeting SR activities should be considered during the development of therapeutics against cancer.

### Control of β-catenin protein level, synthesis versus degradation

β-Catenin is the key mediator of Wnt signalling, and its accumulation is found in a wide spectrum of solid tumours. Its normal function implicates the regulation of self-renewal of tissue stem cells. However, once out of control and ectopically accumulated, β-catenin triggers cancerous cell proliferation, promotes anti-apoptotic activities and induces telomerase expression, all leading to tumour development (Chen et al, 2001; Hoffmeyer et al, 2012; Shkreli et al, 2012; Ueda et al, 2011; Verma et al, 2003). The key question therefore focuses on the understanding of mechanisms that are utilized to control cellular β-catenin protein level. Much is known about the regulation of β-catenin degradation, while relatively little is known about its protein synthesis. In 2003, HuR, a RNA binding protein, was shown to stabilize β-catenin mRNA, and once overexpressed enhances β-catenin production. This activity contributed to proliferation of colon cancer cells (Lopez de Silanes et al, 2003). In contrast to HuR, KSRP was reported to de-stabilize β-catenin mRNA and provide a platform on which both Wnt stimulation and the PI3K/AKT pathway could trigger β-catenin accumulation by counteracting the activity of KSRP (Bikkavilli & Malbon, 2010; Gherzi et al, 2006; Ruggiero et al, 2007). Yet, in a third mode, Quaking was suggested to repress β-catenin mRNA translation (Yang et al, 2010), further complicating the regulation at the level of β-catenin mRNA. Besides RNA binding proteins, eIF6, an essential factor for ribosome biogenesis, has also been found to negatively regulate β-catenin production via a mechanism that remains unclear (Ji et al, 2008). In this study, we proposed that β-catenin mRNA is also under the control of SR proteins, being captured and facilitated toward translational machinery, though mRNA levels were not dramatically altered upon SR overexpression. All these studies together clearly indicate that the regulation at the level of β-catenin protein production is complex and further characterization is needed.

It is generally believed that Wnt stimulation blocks β-catenin degradation and consequently allows accumulation of newly synthesized β-catenin protein (Clevers & Nusse, 2012). In 2010, a new model was proposed in which Wnt3a-stimulated β-catenin accumulation was suggested to be the result of β-catenin mRNA stabilization (Bikkavilli & Malbon, 2010). In either case, synthesis of β-catenin protein is absolutely required for signalling, and theoretically slowing-down protein synthesis should weaken β-catenin accumulation in physiological as well as pathological conditions. Regarding protein synthesis, it was reported that Wnt could activate mTOR via GSK3 inhibition, but whether β-catenin protein translation was stimulated by Wnt was not firmly tested (Inoki et al, 2006). In this study, we show that SR proteins are generally involved in β-catenin accumulation and once overexpressed, they are able to elevate β-catenin protein production. Considering that SRSF3 is a transcriptional target of Wnt/β-catenin signalling (Corbo et al, 2012; Goncalves et al, 2008), and SRSF1 is a target of MYC (Das et al, 2012), which itself is also a major transcriptional target of the Wnt signalling (He et al, 1998), we tend to speculate that a positive feedback loop exists between Wnt/β-catenin signalling and some of the SR proteins.

The contribution of SR proteins on β-catenin accumulation is not as strong as that from impairment of degradation. However, as suggested by Goentoro and Kirschner using mathematic modelling and experimental testing, cells sense a robust fold-change in response to Wnt stimulation and, more importantly, the Wnt-induced fold-change is sensitive to perturbations in the synthesis rate of β-catenin in the responding cells (Goentoro & Kirschner, 2009). In other words, if a cell has a higher basal level of β-catenin, it would obtain a higher fold-change response to the same Wnt stimuli. The overexpressed SR proteins, with their complex activities on mRNA biogenesis, stability control and protein translation, are good candidates for fulfilling such a role. In cells that have higher expression of SRSF genes, an elevated β-catenin basal level would meet this condition, resulting in the cells becoming super-sensitive to stimuli from the environment or internal mutations and accelerating their transformation. In this sense, reduction in the protein synthesis rate in a cell is of great importance in respect of cancer therapy, and this is beautifully demonstrated in APC mutant mice in which inhibition of mTOR drastically suppressed intestinal polyp formation and reduced mortality (Fujishita et al, 2008).

## MATERIALS AND METHODS

### Cell culture

HEK293T, NIH3T3, RKO, HT29 and SW620 cell lines were maintained in Dulbecco's modified Eagle's medium (DMEM), SW480 cell line was cultured in L15 medium and HCT116 cell line was cultured in Mccoy's 5A medium. All culture medium were supplemented with 10% foetal bovine serum (FBS), penicillin and streptomycin.

### Plasmids, siRNA and shRNA

Human SRSF1 and SRSF9 plasmids were kindly given by Prof. Benoit Chabot (University of Sherbrooke, Canada) and Prof. Javier F. Caceres (Medical Research Council Human Genetics Unit, Western General Hospital, UK). Other human SR plasmids of proteins were bought from Origene (OriGene Technologies, Rockville, MD). The expression constructs were generated using PCR and subcloned into pCS2+ vectors with a N-terminal FLAG tag. SRSF1 and SRSF9 were also subcloned into pEF6/Myc-His vector, which were used for establishing stable NIH3T3 cell lines. Using the QuikChange strategy (Stratagene), we generated the point mutations of SRSF1 and SRSF9. All plasmid constructs were verified by sequencing. siRNA synthesis was performed by GenePharma (Shanghai GenePharma Co., Ltd., China) and the siRNA sequence for human SRSF1 and SRSF9 were as follows, human SRSF1-1: GGAAAGAAGATATGACCTA, SRSF1-2: GAAGTTGGCAGGATTTAAATT; human SRSF9-1: GCTGATGTGCAGAAGGATG, SRSF9-2: GGAATATGCCCTGCGTAAA. Transfection of plasmid and siRNA were performed as described previously (Wang et al, 2010; Zhu et al, 2012). The GIPZ lentiviral shRNA constructs targeting human SRSF1, SRSF9 and mouse β-catenin were ordered from Open Biosystems (Thermo Fisher Scientific, USA), and the catalogue numbers were RHS4531- NM_006924, RHS4531-NM_003769.2 and RMM4532-NM_007614, respectively. Lentivirus production and infection was carried out following the protocol described previously (Li et al, 2012).

### Luciferase reporter assay

Wnt responsive Super-TOPFLASH luciferase reporter assays in HEK293T cells were performed in 96-well plates with triplicate as described previously (Wang et al, 2010). DNA per well used: β-catenin 3 ng, SR proteins 5 ng, Wnt1 15 ng. Notch and TGF-β signalling responsive luciferase reporter assays were carried out using the same protocol, except that pGa981-6 luciferase reporter (Kurooka et al, 1998) and CACG luciferase reporter was used as 30 ng/well. All luciferase activities were normalized to Renilla activity.

### Protein stability assay, cell fractionation and Western blotting

Protein stability assay, cell fractionation and Western blotting were performed as described previously (Liang et al, 2011; Wang et al, 2010). Antibodies used as following: SRSF1 (Santa Cruz sc-73026), SRSF9 (Santa Cruz sc-134036), FLAG (Sigma F3165), Myc (Santa Cruz sc-40), β-catenin (BD Biosciences 610154), non-phospho-β-catenin (Cell signalling #4207), CyclinD1 (MBL MD-17-3S), Phospho-p70 S6 Kinase (Cell signalling #9205), p70 S6 Kinase (Cell signalling #9202).

### RNA-binding protein immunoprecipitation (RIP)

RIP assay was performed using Magna RIP RNA-Binding Protein Immunoprecipitation Kit (Millipore, Bedford, MA) following the instruction of the manufactory. FLAG (Sigma F3165) and SRSF1 (Santa Cruz sc-73026) antibodies were used in RIP assays.

**The paper explained**

**PROBLEM**

Wnt/β-catenin signalling plays significant roles in embryogenesis, tissue homeostasis and tumorigenesis. β-Catenin degradation has been the centre of attention in targeting Wnt signalling-mediated tumorigenesis. However, β-catenin synthesis is undeservedly neglected, which also contributes to β-catenin accumulation in cancer cells. The mechanisms mediating the control of β-catenin synthesis remain largely unknown, an issue we addressed by a family of important RNA binding proteins, SR proteins.

**RESULTS**

In our study, we show that SRSF1 and SRSF9 promote β-catenin accumulation via enhancing β-catenin mRNA translation. Similar with SRSF1, SRSF9 also has oncogenic activity, which at least partially due to promoting β-catenin accumulation. Correspondingly, depletion of either SR proteins reduced colon cancer cell proliferation. Moreover, considering the importance of β-catenin's production and degradation, we demonstrated in principle that promoting degradation and blocking production of β-catenin synergize in reducing cancerous β-catenin level.

**IMPACT**

Our study establishes a novel link between the Wnt/β-catenin signalling and the SR proteins and indicates that some of the SR proteins contribute to β-catenin accumulation. Our results suggest that β-catenin is a major translational target of a subset of SR proteins, mediating their tumorigenic activity. Furthermore, our work demonstrates the importance of β-catenin synthesis during tumour progression and emphasizes that promoting degradation and blockage production of β-catenin protein should be equally considered during therapeutic treatment of Wnt signalling-mediated tumour.

### Sucrose gradient sedimentation

Sucrose gradient sedimentation was carried out as previously described (Sanford et al, 2004) with minor modification. After transfection with empty vectors or SR plasmids in HEK293T cells, the cytoplasmic extracts was loaded on top of an 11 ml 10–45% sucrose gradient and ultracentrifuged for 2 h at 36,000 rpm in a SW41Ti rotor (Beckman). The gradient fractions were obtained and analysed using a Teledyne Isco fractionation system. mRNAs from these fractions were extracted and their abundance were detected using RT-PCR. Primers for human β-catenin are following, forward: CAAGCCACAAGATTACAAGAAACGG, reverse: CCATCAACTGGATAGTCAGCACC.

### MTS cell proliferation assay

HCT116 cells were seeded into 96-well plates with 5000 cell per well and transfected with siRNAs. CellTiter 96® AQueous One Solution Cell Proliferation Assay kit (Promega) was used to determine the number of viable cells in each well. Triplicates were included for each sample. The absorbance at 492 nm was measured using a 96-well plate reader.

### Soft agar assay for anchorage-independent colony formation

NIH3T3 cells were transfected with pEF6/Myc-His-SRSF1 or -SRSF9, selected with blasticidin (10 µg/ml) and a mixture of SRSF1 or SRSF9-over-expressing NIH3T3 cell lines were obtained. 1 × 10^4^ NIH3T3 cells were suspended in 0.3% low melting point agarose and then laid onto a bottom layer containing 1% agarose in six-well plates with triplicate. Colonies were stained and counted 4 weeks later. For SW620 cells, 500 cells were suspended in 0.3% top agarose and laid on top of the 0.6% bottom agarose in six-well plates with triplicate. Colonies were stained and counted 2 weeks later.

### Immunohistochemical analysis

Immunohistochemistry was performed with human colon carcinoma (grades I–III with normal controls) tissue array (BC05118a) and multiple organ tissue array (MC5003a) by Cybrdi, Inc. (Xi'an, China). Anti-SRSF1 (Invitrogen 325600), SRSF9 (Santa Cruz sc-134036) and β-catenin (BD Biosciences 610154) antibodies were used at 1:50, 1:10 and 1:200 dilutions, respectively. A semi-quantitative analysis in stained sections was performed by an independent pathologist.

### Tumour induction in nude mice

BALB/c nude mice were injected subcutaneously with 2.5 × 10^5^ NIH3T3 cells stably expressing pEF6-vectors or stably expressing SR proteins, with eight mice in each group. Tumour size was measured with vernier caliper at Day 7, 13, 16, 19 after implantation, and tumour volume was calculated with the following formula: volume = (width)^2 ^× length/2. Tumour weight was recorded when the mice were received euthanasia at Day 19.

## Author contributions

YF and BH performed most of the experiments with partial contributions from ZS and JHa; ZS, JHa, JHu and YW verified the initial *Xenopus* clones from *Xenopus* cDNA library; YF and WW designed and supervised the study; YF and WW wrote the manuscript with contributions from BH.

## Notes

As a service to our authors and readers, this journal provides supporting information supplied by the authors. Such materials are peer reviewed and may be re-organized for online delivery, but are not copy-edited or typeset. Technical support issues arising from supporting information (other than missing files) should be addressed to the authors.

## References

[b1] Anczukow O, Rosenberg AZ, Akerman M, Das S, Zhan L, Karni R, Muthuswamy SK, Krainer AR (2012). The splicing factor SRSF1 regulates apoptosis and proliferation to promote mammary epithelial cell transformation. Nat Struct Mol Biol.

[b2] Archbold HC, Yang YX, Chen L, Cadigan KM (2012). How do they do Wnt they do?: regulation of transcription by the Wnt/beta-catenin pathway. Acta Physiol (Oxf).

[b3] Ballou LM, Lin RZ (2008). Rapamycin and mTOR kinase inhibitors. J Chem Biol.

[b4] Bikkavilli RK, Malbon CC (2010). Dishevelled-KSRP complex regulates Wnt signaling through post-transcriptional stabilization of beta-catenin mRNA. J Cell Sci.

[b5] Bourgeois CF, Lejeune F, Stevenin J (2004). Broad specificity of SR (serine/arginine) proteins in the regulation of alternative splicing of pre-messenger RNA. Prog Nucleic Acid Res Mol Biol.

[b6] Caceres JF, Misteli T, Screaton GR, Spector DL, Krainer AR (1997). Role of the modular domains of SR proteins in subnuclear localization and alternative splicing specificity. J Cell Biol.

[b7] Chen S, Guttridge DC, You Z, Zhang Z, Fribley A, Mayo MW, Kitajewski J, Wang CY (2001). Wnt-1 signaling inhibits apoptosis by activating beta-catenin/T cell factor-mediated transcription. J Cell Biol.

[b8] Chien AJ, Conrad WH, Moon RT (2009). A Wnt survival guide: from flies to human disease. J Invest Dermatol.

[b9] Clevers H (2006). Wnt/beta-catenin signaling in development and disease. Cell.

[b10] Clevers H, Nusse R (2012). Wnt/beta-catenin signaling and disease. Cell.

[b11] Corbo C, Orru S, Gemei M, Noto RD, Mirabelli P, Imperlini E, Ruoppolo M, Vecchio LD, Salvatore F (2012). Protein cross-talk in CD133+ colon cancer cells indicates activation of the Wnt pathway and upregulation of SRp20 that is potentially involved in tumorigenicity. Proteomics.

[b12] Das S, Anczukow O, Akerman M, Krainer AR (2012). Oncogenic splicing factor SRSF1 is a critical transcriptional target of MYC. Cell Rep.

[b13] Davidson G, Wu W, Shen J, Bilic J, Fenger U, Stannek P, Glinka A, Niehrs C (2005). Casein kinase 1 gamma couples Wnt receptor activation to cytoplasmic signal transduction. Nature.

[b14] de Sousa EM, Vermeulen L, Richel D, Medema JP (2011). Targeting Wnt signaling in colon cancer stem cells. Clin Cancer Res.

[b15] Ezponda T, Pajares MJ, Agorreta J, Echeveste JI, Lopez-Picazo JM, Torre W, Pio R, Montuenga LM (2010). The oncoprotein SF2/ASF promotes non-small cell lung cancer survival by enhancing survivin expression. Clin Cancer Res.

[b16] Fujishita T, Aoki K, Lane HA, Aoki M, Taketo MM (2008). Inhibition of the mTORC1 pathway suppresses intestinal polyp formation and reduces mortality in ApcDelta716 mice. Proc Natl Acad Sci USA.

[b17] Gherzi R, Trabucchi M, Ponassi M, Ruggiero T, Corte G, Moroni C, Chen CY, Khabar KS, Andersen JS, Briata P (2006). The RNA-binding protein KSRP promotes decay of beta-catenin mRNA and is inactivated by PI3K-AKT signaling. PLoS Biol.

[b18] Goentoro L, Kirschner MW (2009). Evidence that fold-change, and not absolute level, of beta-catenin dictates Wnt signaling. Mol Cell.

[b19] Goncalves V, Matos P, Jordan P (2008). The beta-catenin/TCF4 pathway modifies alternative splicing through modulation of SRp20 expression. RNA.

[b20] Graveley BR (2000). Sorting out the complexity of SR protein functions. RNA.

[b21] Hannoush RN (2008). Kinetics of Wnt-driven beta-catenin stabilization revealed by quantitative and temporal imaging. PLoS ONE.

[b22] Hart M, Concordet JP, Lassot I, Albert I, del los Santos R, Durand H, Perret C, Rubinfeld B, Margottin F, Benarous R (1999). The F-box protein beta-TrCP associates with phosphorylated beta-catenin and regulates its activity in the cell. Curr Biol.

[b23] He TC, Sparks AB, Rago C, Hermeking H, Zawel L, da Costa LT, Morin PJ, Vogelstein B, Kinzler KW (1998). Identification of c-MYC as a target of the APC pathway. Science.

[b24] Hertel KJ, Graveley BR (2005). RS domains contact the pre-mRNA throughout spliceosome assembly. Trends Biochem Sci.

[b25] Hoffmeyer K, Raggioli A, Rudloff S, Anton R, Hierholzer A, Del Valle I, Hein K, Vogt R, Kemler R (2012). Wnt/beta-catenin signaling regulates telomerase in stem cells and cancer cells. Science.

[b26] Huang SM, Mishina YM, Liu S, Cheung A, Stegmeier F, Michaud GA, Charlat O, Wiellette E, Zhang Y, Wiessner S (2009). Tankyrase inhibition stabilizes axin and antagonizes Wnt signalling. Nature.

[b27] Huang Y, Steitz JA (2005). SRprises along a messenger's journey. Mol Cell.

[b28] Inoki K, Ouyang H, Zhu T, Lindvall C, Wang Y, Zhang X, Yang Q, Bennett C, Harada Y, Stankunas K (2006). TSC2 integrates Wnt and energy signals via a coordinated phosphorylation by AMPK and GSK3 to regulate cell growth. Cell.

[b29] Jamieson C, Sharma M, Henderson BR (2012). Wnt signaling from membrane to nucleus: beta-catenin caught in a loop. Int J Biochem Cell Biol.

[b30] Ji Y, Shah S, Soanes K, Islam MN, Hoxter B, Biffo S, Heslip T, Byers S (2008). Eukaryotic initiation factor 6 selectively regulates Wnt signaling and beta-catenin protein synthesis. Oncogene.

[b31] Jia R, Li C, McCoy JP, Deng CX, Zheng ZM (2010). SRp20 is a proto-oncogene critical for cell proliferation and tumor induction and maintenance. Int J Biol Sci.

[b32] Jin LH, Shao QJ, Luo W, Ye ZY, Li Q, Lin SC (2003). Detection of point mutations of the Axin1 gene in colorectal cancers. Int J Cancer.

[b33] Karni R, de Stanchina E, Lowe SW, Sinha R, Mu D, Krainer AR (2007). The gene encoding the splicing factor SF2/ASF is a proto-oncogene. Nat Struct Mol Biol.

[b34] Karni R, Hippo Y, Lowe SW, Krainer AR (2008). The splicing-factor oncoprotein SF2/ASF activates mTORC1. Proc Natl Acad Sci USA.

[b35] Kentsis A, Topisirovic I, Culjkovic B, Shao L, Borden KL (2004). Ribavirin suppresses eIF4E-mediated oncogenic transformation by physical mimicry of the 7-methyl guanosine mRNA cap. Proc Natl Acad Sci USA.

[b36] Klaus A, Birchmeier W (2008). Wnt signalling and its impact on development and cancer. Nat Rev Cancer.

[b37] Kurooka H, Kuroda K, Honjo T (1998). Roles of the ankyrin repeats and C-terminal region of the mouse notch1 intracellular region. Nucleic Acids Res.

[b38] Li Z, Fei T, Zhang J, Zhu G, Wang L, Lu D, Chi X, Teng Y, Hou N, Yang X (2012). BMP4 signaling acts via dual-specificity phosphatase 9 to control ERK activity in mouse embryonic stem cells. Cell Stem Cell.

[b39] Liang J, Fu Y, Cruciat CM, Jia S, Wang Y, Tong Z, Tao Q, Ingelfinger D, Boutros M, Meng A (2011). Transmembrane protein 198 promotes LRP6 phosphorylation and Wnt signaling activation. Mol Cell Biol.

[b40] Liu C, Kato Y, Zhang Z, Do VM, Yankner BA, He X (1999). Beta-Trcp couples beta-catenin phosphorylation-degradation and regulates Xenopus axis formation. Proc Natl Acad Sci USA.

[b41] Liu C, Li Y, Semenov M, Han C, Baeg GH, Tan Y, Zhang Z, Lin X, He X (2002). Control of beta-catenin phosphorylation/degradation by a dual-kinase mechanism. Cell.

[b42] Logan CY, Nusse R (2004). The Wnt signaling pathway in development and disease. Annu Rev Cell Dev Biol.

[b43] Long JC, Caceres JF (2009). The SR protein family of splicing factors: master regulators of gene expression. Biochem J.

[b44] Lopez de Silanes I, Fan J, Yang X, Zonderman AB, Potapova O, Pizer ES, Gorospe M (2003). Role of the RNA-binding protein HuR in colon carcinogenesis. Oncogene.

[b45] MacDonald BT, Tamai K, He X (2009). Wnt/beta-catenin signaling: components, mechanisms, and diseases. Dev Cell.

[b46] Manley JL, Krainer AR (2010). A rational nomenclature for serine/arginine-rich protein splicing factors (SR proteins). Genes Dev.

[b47] Manley JL, Tacke R (1996). SR proteins and splicing control. Genes Dev.

[b48] Meric F, Hunt KK (2002). Translation initiation in cancer: a novel target for therapy. Mol Cancer Ther.

[b49] Michlewski G, Sanford JR, Caceres JF (2008). The splicing factor SF2/ASF regulates translation initiation by enhancing phosphorylation of 4E-BP1. Molecular cell.

[b50] Morin PJ, Sparks AB, Korinek V, Barker N, Clevers H, Vogelstein B, Kinzler KW (1997). Activation of beta-catenin-Tcf signaling in colon cancer by mutations in beta-catenin or APC. Science.

[b51] Mosimann C, Hausmann G, Basler K (2009). Beta-catenin hits chromatin: regulation of Wnt target gene activation. Nat Rev Mol Cell Biol.

[b52] Philipps D, Celotto AM, Wang QQ, Tarng RS, Graveley BR (2003). Arginine/serine repeats are sufficient to constitute a splicing activation domain. Nucleic Acids Res.

[b53] Polakis P (2007). The many ways of Wnt in cancer. Curr Opin Genet Dev.

[b54] Polakis P (2012a). Drugging Wnt signalling in cancer. EMBO J.

[b55] Polakis P (2012b). Wnt signaling in cancer. Cold Spring Harb Perspect Biol.

[b56] Rubinfeld B, Souza B, Albert I, Muller O, Chamberlain SH, Masiarz FR, Munemitsu S, Polakis P (1993). Association of the APC gene product with beta-catenin. Science.

[b57] Ruggiero T, Trabucchi M, Ponassi M, Corte G, Chen CY, al-Haj L, Khabar KS, Briata P, Gherzi R (2007). Identification of a set of KSRP target transcripts upregulated by PI3K-AKT signaling. BMC Mol Biol.

[b58] Salic A, Lee E, Mayer L, Kirschner MW (2000). Control of beta-catenin stability: reconstitution of the cytoplasmic steps of the wnt pathway in Xenopus egg extracts. Mol Cell.

[b59] Sanford JR, Gray NK, Beckmann K, Caceres JF (2004). A novel role for shuttling SR proteins in mRNA translation. Genes Dev.

[b60] Schepers A, Clevers H (2012). Wnt signaling, stem cells, and cancer of the gastrointestinal tract. Cold Spring Harb Perspect Biol.

[b61] Schwarz-Romond T, Fiedler M, Shibata N, Butler PJ, Kikuchi A, Higuchi Y, Bienz M (2007). The DIX domain of Dishevelled confers Wnt signaling by dynamic polymerization. Nat Struct Mol Biol.

[b62] Screaton GR, Caceres JF, Mayeda A, Bell MV, Plebanski M, Jackson DG, Bell JI, Krainer AR (1995). Identification and characterization of three members of the human SR family of pre-mRNA splicing factors. EMBO J.

[b63] Shepard PJ, Hertel KJ (2009). The SR protein family. Genome Biol.

[b64] Shkreli M, Sarin KY, Pech MF, Papeta N, Chang W, Brockman SA, Cheung P, Lee E, Kuhnert F, Olson JL (2012). Reversible cell-cycle entry in adult kidney podocytes through regulated control of telomerase and Wnt signaling. Nat Med.

[b65] Shultz JC, Goehe RW, Murudkar CS, Wijesinghe DS, Mayton EK, Massiello A, Hawkins AJ, Mukerjee P, Pinkerman RL, Park MA (2011). SRSF1 regulates the alternative splicing of caspase 9 via a novel intronic splicing enhancer affecting the chemotherapeutic sensitivity of non-small cell lung cancer cells. Mol Cancer Res.

[b66] Silvera D, Formenti SC, Schneider RJ (2010). Translational control in cancer. Nat Rev Cancer.

[b67] Su Y, Fu C, Ishikawa S, Stella A, Kojima M, Shitoh K, Schreiber EM, Day BW, Liu B (2008). APC is essential for targeting phosphorylated beta-catenin to the SCFbeta-TrCP ubiquitin ligase. Mol Cell.

[b68] Taya S, Yamamoto T, Kanai-Azuma M, Wood SA, Kaibuchi K (1999). The deubiquitinating enzyme Fam interacts with and stabilizes beta-catenin. Genes Cells.

[b69] Twyffels L, Gueydan C, Kruys V (2011). Shuttling SR proteins: more than splicing factors. FEBS J.

[b70] Ueda Y, Hiyama E, Kamimatsuse A, Kamei N, Ogura K, Sueda T (2011). Wnt signaling and telomerase activation of hepatoblastoma: correlation with chemosensitivity and surgical resectability. J Pediatr Surg.

[b71] Valenta T, Hausmann G, Basler K (2012). The many faces and functions of beta-catenin. EMBO J.

[b72] van Amerongen R, Nusse R (2009). Towards an integrated view of Wnt signaling in development. Development.

[b73] Verma UN, Surabhi RM, Schmaltieg A, Becerra C, Gaynor RB (2003). Small interfering RNAs directed against beta-catenin inhibit the in vitro and in vivo growth of colon cancer cells. Clin Cancer Res.

[b74] Waaler J, Machon O, von Kries JP, Wilson SR, Lundenes E, Wedlich D, Gradl D, Paulsen JE, Machonova O, Dembinski JL (2011). Novel synthetic antagonists of canonical Wnt signaling inhibit colorectal cancer cell growth. Cancer Res.

[b75] Wang Y, Fu Y, Gao L, Zhu G, Liang J, Gao C, Huang B, Fenger U, Niehrs C, Chen YG (2010). Xenopus skip modulates Wnt/beta-catenin signaling and functions in neural crest induction. J Biol Chem.

[b76] Wang L, Heidt DG, Lee CJ, Yang H, Logsdon CD, Zhang L, Fearon ER, Ljungman M, Simeone DM (2009). Oncogenic function of ATDC in pancreatic cancer through Wnt pathway activation and beta-catenin stabilization. Cancer Cell.

[b77] Xing Y, Clements WK, Kimelman D, Xu W (2003). Crystal structure of a beta-catenin/axin complex suggests a mechanism for the beta-catenin destruction complex. Genes Dev.

[b78] Yang G, Fu H, Zhang J, Lu X, Yu F, Jin L, Bai L, Huang B, Shen L, Feng Y (2010). RNA-binding protein quaking, a critical regulator of colon epithelial differentiation and a suppressor of colon cancer. Gastroenterology.

[b79] Yoshino H, Enokida H, Chiyomaru T, Tatarano S, Hidaka H, Yamasaki T, Gotannda T, Tachiwada T, Nohata N, Yamane T (2012). Tumor suppressive microRNA-1 mediated novel apoptosis pathways through direct inhibition of splicing factor serine/arginine-rich 9 (SRSF9/SRp30c) in bladder cancer. Biochem Biophys Res Commun.

[b80] Zhong XY, Wang P, Han J, Rosenfeld MG, Fu XD (2009). SR proteins in vertical integration of gene expression from transcription to RNA processing to translation. Mol Cell.

[b81] Zhu G, Wang Y, Huang B, Liang J, Ding Y, Xu A, Wu W (2012). A Rac1/PAK1 cascade controls beta-catenin activation in colon cancer cells. Oncogene.

