# Supplementary material for: SRSF1 and SRSF9 RNA binding proteins promote Wnt signalling-mediated tumorigenesis by enhancing β-catenin biosynthesis
Source: EMBO Mol Med. 2013 Apr 17;5(5):737–50. doi: 10.1002/emmm.201202218 (PMC3662316; doi:10.1002/emmm.201202218)
Supplement: Supplementary file 1 [file emmm0005-0737-sd1.pdf]

EMBO
Molecular Medicine

# SRSF1 and SRSF9 RNA binding proteins promote Wnt signaling-mediated tumorigenesis by enhancing β-catenin biosynthesis

Yu Fu, Binlu Huang, Zhen Shi, Jiayu Han, Ying Wang, Jieqiong Huangfu and Wei Wu

*Corresponding author: Wei Wu, Tsinghua University*

**Review timeline:**

| | |
|---|---|
| Submission date: | 28 October 2012 |
| Editorial Decision: | 03 January 2013 |
| Revision received: | 08 February 2013 |
| Accepted: | 08 March 2013 |

## Transaction Report:

(Note: With the exception of the correction of typographical or spelling errors that could be a source of ambiguity, letters and reports are not edited. The original formatting of letters and referee reports may not be reflected in this compilation.)

*Editor: Roberto Buccione*

1st Editorial Decision 03 January 2013

Thank you for the submission of your manuscript to EMBO Molecular Medicine. I am sorry that it has taken so long to get back to you on your manuscript.

In this case we experienced unusual difficulties in securing three appropriate reviewers. Further to this, while Reviewers 1 and 3 delivered their evaluations in a timely manner we did not receive the third Reviewer's input. As the evaluations from the first two Reviewers are consistent, and a further delay cannot be justified, I have decided to proceed based on these evaluations. I should inform you, however, that if in the meanwhile, we should receive the third review and only if it raises significant caveats, these would need to be taken into consideration. We would not, however, ask you to comply with any further-reaching requests.

You will see that the Reviewers are generally supportive but also raise significant issues that prevent us from considering publication at this time.

Reviewer 1 notes that nuclear amounts of beta-catenin are barely modified by alterations of SR proteins; s/he would like to understand why and also asks how efficiently is nuclear beta-catenin detected by your fractionation scheme. Reviewer 1 also wonders why Srsf1 and Srsf9 knockdown have similar effects on reporter activity but not on beta-catenin; s/he suggests a combined knockdown experiment to address the issue. In general Reviewer 1 also suggests a number of other interventions, which in my opinion are also important, both at the experimental level and on the presentation of the figures and text that are important to improve clarity, readability and accuracy.

Reviewer 3 notes that p70S6k phosphorylation data are unclear and suggests that a complete analysis of SRSF would help to determine if the two groups of proteins have similar differences in

the mTOR pathway. S/he also expresses concern that the reduction of beta-catenin by SRSF knockdown is mild and that it is thus important to establish whether cell proliferation effects are observed. Similarly to Reviewer 1, Reviewer 3 also suggests a number of other interventions, both at the experimental level and on the presentation of the figures and text.

Finally, both Reviewers are concerned about the quality and thus significance of the immunohistochemistry staining in Fig. 4 and challenge your interpretation that SRSF9 is more enriched in the cytoplasm than the nucleus, compared to normal tissues. While publication of the paper cannot be considered at this stage, we would be pleased to consider a suitably revised submission, provided, however, that the Reviewers' concerns are fully addressed with additional experimental data where appropriate.

Please note that it is EMBO Molecular Medicine policy to allow a single round of revision only and that, therefore, acceptance or rejection of the manuscript will depend on the completeness of your responses included in the next, final version of the manuscript.

As you know, EMBO Molecular Medicine has a "scooping protection" policy, whereby similar findings that are published by others during review or revision are not a criterion for rejection. However, I do ask you to get in touch with us after three months if you have not completed your revision, to update us on the status. Please also contact us as soon as possible if similar work is published elsewhere.

I look forward to seeing a revised form of your manuscript as soon as possible.

***** Reviewer's comments *****

Referee #1:

The authors show that mRNA binding factors of the Srsf protein family increase beta-catenin levels probably by mediating interaction of beta-catenin mRNA to the translational machinery including mTOR signalling. It is further shown that Srsf1 and 9 can transform NIH3T3 cells which is in part dependent on beta-catenin, and that these factors are required for the transformed phenotype of SW620 colon carcinoma cells. Inhibition of translation by interfering with mTOR combined with increased degradation is proposed as a strategy to combat tumorigenic beta-catenin in cancer. To consider beta-catenin production in parallel to its degradation as a way to regulate beta-catenin levels in Wnt signalling is a rather novel approach and well documented here. The results are mostly clear-cut and presented in excellent figures based on solid experimentation. Mechanistically, the Srsf action remains unclear, though because the connection between mRNA binding and mTOR activation to promote beta-catenin translation is somewhat vague. It cannot be ruled out from the data that Srsfs act indirectly, e.g. by altering other components of the Wnt pathway that impact on beta-catenin synthesis but this is obviously a topic for subsequent investigations. It's mainly some details that should be dealt with at the revision stage.

1. Nuclear amounts of beta-catenin vary only slightly, if at all, with alterations of SR proteins in Figs. 1D, 5A and D. Is that due to inefficiency of nuclear extraction or does it mean that Sr proteins mainly affect cytoplasmic beta-catenin? How well is nuclear beta-catenin seen in Wnt stimulated cells using this fractionation scheme? Moreover, markers for lamin and tubulin markers should be shown in both fractions to determine their purity.

2. In Fig.3A knockdown of both Srsf1 and Srsf9 has similar consequences on reporter activity whereas knockdown of Srfs9 is much less efficient in downregulating beta-catenin. How can these differences be explained? Would combined knockdown of both Srsfs lead to stronger effects? Also, Fig 3C and E need quantification of several independent experiments to support effects on beta-catenin levels. In Fig. 3D it seems that only the upper band of axin2 is affected. Is the lower band a background band? Does it remain with siRNA to axin2? If it is axin2 could it be that it reflects a splice variant and that axin2 splicing is affected by Srsfs? With respect to this panel, it is not mentioned which antibodies were used to detect axin2 and also cyclinD1.

3. Fig. 5A,B,D: panels labelled with "myc" should be relabelled "myc-SR", similar to "FLAG-SR" in Fig. 1C to avoid the impression that the c-myc protein was analysed. Fig. 1C,D should show

consistent labelling of "FLAG-SR". In Fig. 3 legend should state that two different siRNAs were used for each Srsf1 and Srsf9 and also which of the two was used in Fig. 3A.

4. What is the purpose of showing the ABC antibody western blot fig. 7? It is not mentioned in the main text, nor was it used in the other figures analysing beta-catenin.

5. What is the purpose of showing Srsf western blot of the different colon carcinoma cells?

6. The immunohistochemical staining in Fig. 4A is not fully satisfactory. More importantly, the statement that SRSF9 is more enriched in cytoplasm than in nucleus as compared to normal tissues is not reflected by the pictures. In fact this seems only the case for skin tumors whereas in the other tumor sections the protein seems to very strongly present in the nucleus. This statement should be deleted or supported by more clear data. What is the strong brown staining in normal brain? Which anatomical part of the brain is shown? It should be also stated what types of tumors are actually shown.

Referee #3:

Fu and colleagues provide intriguing data that a subset of SRSF proteins regulate beta-catenin RNA stability and protein accumulation. The studies are well controlled and conducted in appropriate cell lines using both overexpression and knockdown systems. Major and minor concerns are listed below:

The authors show that cytoplasmic b-catenin is accumulated but not nuclear form after SRSF overexpression in Figure 1. This is an important point given that nuclear b-catenin mediates its oncogenic activity. This should be checked in colon cancer cell lines as well where the authors show SRSF inhibition of b-catenin pathway.

Authors suggest that SRSF1 and SRSF9 promote beta-catenin accumulation in a mTOR dependent mechanism (Figure 2E). The p-S6K kinase activity does not correlate too well as SRSF9 shows more S6K phospohorylation (Figure 2E) but less robust beta-catenin accumulation (Fig. 1C). A complete analysis of SRSF proteins (1-12) would be informative in drawing a more significant conclusion as to whether the two groups of SRSF proteins (1,3,5,7,8,9,10,12 vs 2,4,6,11) show similar differences in mTOR pathway activity.

The authors' conclusion on page 11, line 5 is overstated. It is not clear from this data that these effects are "mTOR-dependent". To make this conclusion more decisive, further data should be provide - e..g using mTOR inhibitors in the presence/absence of SRSF1/9 to determine whether b-catenin accumulation is blocked.

Authors show intriguing data that colon cancer cell lines reduce b-catenin levels after SRSF1/9 siRNA inhibition. Published data shows that b-catenin active cell lines such as HCT-116 and SW480 are dependent on b-catenin activity for proliferation. Since the ability of SRSF RNAi to reduc b-catenin protein appears mild (Figure 3D-E), it is important to know whether effects on cell proliferation were seen in these models. It is not clear why soft agar colony assays were used as a read-out in Figure 6.

The immunohistochemical staining shows that this antibody is non-specific and displays background signal. For example, strong keratin staining is seen in the epidermis. The fact that in some tissues cytoplasmic staining is seen but in other nuclear may reflect non-specific staining rather than target distribution changes as the authors suggest. The authors should provide validation and supporting evidence that this antibody is specifically detecting SRSF9 in tissues. For example, do RNA based studies (microarray or qPCR based) confirm SRSF9 overexpression in the tumor type indicated? Does the IHC signal dissapear in cell line pellets after SRSF9 knockdown ?

Page 14, 2nd paragraph: the correlation between b-catenin overexpression and SRSF overexpression does not appear to be statistically significant as the same percent of tumors were b-catenin high

regardless of SRSF1 expression. The authors present data showing that many SRSF proteins can mediate b-catenin accumulation so does it make sense that only one SRSF protein will correlate to its expression in colon cancer ? A more robust bio-informatic analysis of SRSF protein expression and b-catenin expression would be required beyond what is presented here.

Minor points:

Page 12, 2nd paragraph, the data focuses on SRSF9 but authors write "....and SRSF1 has been suggested....)

Authors interchange SRSF and SRSP (page 12, second to last line).

Figure 4B: the SRSF1 blot is massively over-exposed and impossible to determine the degree of exogenous SRSF1 overexpression compared to endogenous levels.

Figure 5F : the resolution of the images provided is low. Higher resolution photos should be provided.

1st Revision - authors' response 08 February 2013

Response to Referees

Referee #1:

*The authors show that mRNA binding factors of the Srsf protein family increase beta-catenin levels probably by mediating interaction of beta-catenin mRNA to the translational machinery including mTOR signalling. It is further shown that Srsf1 and 9 can transform NIH3T3 cells which is in part dependent on beta-catenin, and that these factors are required for the transformed phenotype of SW620 colon carcinoma cells. Inhibition of translation by interfering with mTOR combined with increased degradation is proposed as a strategy to combat tumorigenic beta-catenin in cancer. To consider beta-catenin production in parallel to its degradation as a way to regulate beta-catenin levels in Wnt signalling is a rather novel approach and well documented here. The results are mostly clear-cut and presented in excellent figures based on solid experimentation. Mechanistically, the Srsf action remains unclear, though because the connection between mRNA binding and mTOR activation to promote beta-catenin translation is somewhat vague. It cannot be ruled out from the data that Srsfs act indirectly, e.g. by altering other components of the Wnt pathway that impact on beta-catenin synthesis but this is obviously a topic for subsequent investigations. It's mainly some details that should be dealt with at the revision stage.*

Thanks for your support. We agree that it is impossible at this moment to rule out possibilities that SRSF proteins may affect other components of the Wnt pathway. It will be investigated in the future.

*1. Nuclear amounts of beta-catenin vary only slightly, if at all, with alterations of SR proteins in Figs. 1D, 5A and D. Is that due to inefficiency of nuclear extraction or does it mean that Sr proteins mainly affect cytoplasmic beta-catenin? How well is nuclear beta-catenin seen in Wnt stimulated cells using this fractionation scheme? Moreover, markers for lamin and tubulin markers should be shown in both fractions to determine their purity.*

The cell fractionation and nuclear extraction were performed following published protocols (Wu et al, 2008) and seemed to be working fine. As shown in Referee Figure 1, Wnt3a treatment induced massive beta-catenin accumulation in both nuclear and cytosol fractions. Both fractions are also pure enough as indicated by Lamin A/C or Tubulin, respectively. The two markers are included in other fractionation experiments showing in the revised manuscript.

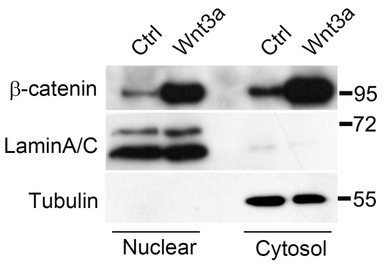


Referee Figure 1, cell fractionation after control or Wnt3a treatment in HEK293T cells.

Regarding SR over-expression-induced beta-catenin accumulation, we have a handful (4-5) repeats of each experiments and the results are splitted. Some showed a slight higher level of beta-catenin in the nuclear fraction, while the others not. In contrast, the enhanced cytosolic beta-catenin accumulation was constantly observed. In this situation, we have to conclude that, upon SR over-expression, only the cytosolic beta-catenin pool is significantly elevated.

Beta-catenin nuclear accumulation is a complex process under stringent regulation. It has been reported by several labs that Wnt stimulation not only triggers the stabilization and accumulation of beta-catenin protein, but also causes, via PI3K/Rac/JNK cascade, modifications of beta-catenin, e.g., phosphorylations, that enhances its nuclear translocation (Hu & Li, 2010; Tenbaum et al, 2012; Wu et al, 2008). These modifications could also be triggered, independent of Wnt ligand, by Ras/MAPK pathway as well, which are hyper-active in tumour cells (Guardavaccaro & Clevers, 2012; Janssen et al, 2006). In this regard, SR-induced beta-catenin should, at least in cells which have low level of PI3K/Rac/JNK and Ras/MAPK signalling, stay mainly in the cytosol since it lacks the required modifications for nuclear transportation/retention. Of course this is not to say that these beta-catenin proteins will only stay in the cytosol since their distribution could be influenced by local environment such as growth factors from surrounding cells. This is also consistent with the observation that in HCT116 cells, enhanced beta-catenin accumulation was observed in both fractions (Referee Figure 2), since in these cancer cells, the PI3K/Rac/JNK or RAS/MAPK pathways are hyper-active.

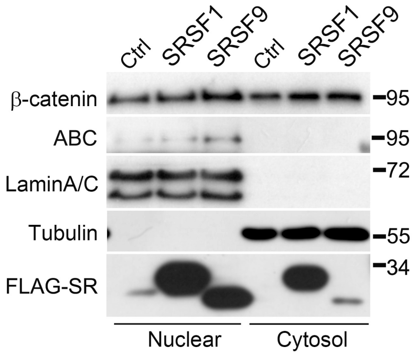


Referee Figure 2, fractionation of HCT116 cells over-expressing SR proteins.

To further clarify this issue, we detected the non-phosphorylated form, the so-called active form, of beta-catenin using the ABC antibody in these cell fractions. It seems that this active form of beta-catenin is slightly but constantly higher in the nuclear fraction after SR over-expression (Fig. 1D, 5A, 5B, Referee Figure 2). These results suggest that SR over-expression promotes cytosolic accumulation of beta-catenin and this may further leads to elevation of the transcriptional active form of beta-catenin in the cell nucleus.

Moreover, it is reported recently by Piccolo and colleagues (Azzolin et al, 2012) that beta-catenin has a cytosolic function in mediating stability control of TAZ, which is also tumorigenic. Although, in that paper it was not shown whether elevation of beta-catenin level could enhance TAZ accumulation, according to their working model, this is likely to happen. When beta-catenin level reaches the threshold exceeding Trcp-mediated degradation, it will likely protect TAZ from degradation. In this case, TAZ may mediate some of the cell transformation activity through cytosolic accumulated beta-catenin.

Finally, we have to accept that SR-induced beta-catenin accumulation is much less than that from Wnt stimulation or APC mutation. Therefore, we believe that beta-catenin is just one of the key target proteins involved in SR-over-expression-induced cell transformation. Our loss-of-function results (Fig.5C) also indicated that knock-down beta-catenin just partially reduced cell transformation efficacy by SR proteins. These targets, including BIN1, MNK2, survivin, beta-catenin and other unidentified ones, synergistically promote tumourigenesis in SR-over-expression cells.

*2. In Fig.3A knockdown of both Srsf1 and Srsf9 has similar consequences on reporter activity whereas knockdown of Srfs9 is much less efficient in down regulating beta-catenin. How can these differences be explained? Would combined knockdown of both Srsfs lead to stronger effects? Also, Fig 3C and E need quantification of several independent experiments to support effects on beta-catenin levels.*

Reporter assays (Fig. 3A) were performed in HEK293T cells, while the beta-catenin measurements (Fig. 3C, E) were performed in RKO and HCT116 cells respectively. Since Srsf genes are differentially expressed in different tissues or cell lines, their contribution to beta-catenin synthesis may be different. At the moment we cannot directly compare the absolute levels of SR proteins in these cell lines. It is quite possible that the protein level of SRSF1 is higher than that of SRSF9 in RKO and HCT116 cells, therefore, it contributes more to beta-catenin synthesis than SRSF9 does.

As suggested, we performed the double knockdown experiments and indeed observed further down-regulation of beta-catenin protein level in HCT116 cells (Referee Figure 3).

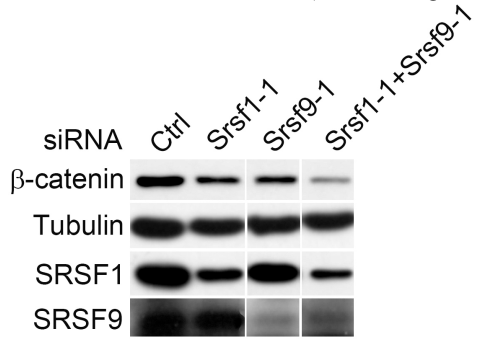


Referee Figure 3, combined knockdown of Srsf1 and Srsf9 caused further down-regulation of beta-catenin protein level in HCT116 cells. This figure is assembled with bands cut from the same X-ray film.

Quantifications of Fig. 3C and 3E were included as suggested.

*3, In Fig. 3D it seems that only the upper band of axin2 is affected. Is the lower band a background band? Does it remain with siRNA to axin2? If it is axin2 could it be that it reflects a splice variant and that axin2 splicing is affected by Srsfs? With respect to this panel, it is not mentioned which antibodies were used to detect axin2 and also cyclinD1.*

Axin2 was only detected here as a target of Wnt signalling, we mistakenly considered the lower band as a non-specific one. When the previous results were re-evaluated, we realized that these two bands are both specific and both bands were down-regulated in two other experiments. The two bands normally localized very close to each other that sometimes are seen as one band. In this particular experiment, the two bands were well separated probably because the gel was running too long. Since it is not absolutely required for this study, we decided to remove the Axin2 result. NCBI database search identifies no alternative splicing variant of human Axin2 gene and our preliminary result indicated that the upper band is not a phosphorylated form (data not shown). It is indeed an interesting question that why Axin2 has this two band pattern. Further investigation is needed.

*4. Fig. 5A,B,D: panels labelled with "myc" should be relabelled "myc-SR", similar to "FLAG-SR" in Fig. 1C to avoid the impression that the c-myc protein was analysed. Fig. 1C,D should show consistent labelling of "FLAG-SR". In Fig. 3 legend should state that two different siRNAs were*

*used for each Srsf1 and Srsf9 and also which of the two was used in Fig. 3A.*

Corrected.

*5. What is the purpose of showing the ABC antibody western blot fig. 7? It is not mentioned in the main text, nor was it used in the other figures analysing beta-catenin.*

ABC antibody recognizes non-phosphorylated beta-catenin, which is believed as the active form. It was used to show that the real transcriptional active beta-catenin is also reduced by the treatments. This is now mentioned in the main text and this antibody is also used to detect SR-enhanced active beta-catenin in Fig. 1D, 5A, 5B.

*6. What is the purpose of showing Srsf western blot of the different colon carcinoma cells?*

In order to choose proper cell lines for knockdown experiments, we measured the levels of SR proteins in several cancer cell lines. This could have been mentioned as “data not shown”, but due to EMM’s policy, “data not shown” is not allowed.

*7. The immunohistochemical staining in Fig. 4A is not fully satisfactory. More importantly, the statement that SRSF9 is more enriched in cytoplasm than in nucleus as compared to normal tissues is not reflected by the pictures. In fact this seems only the case for skin tumours whereas in the other tumour sections the protein seems to very strongly present in the nucleus. This statement should be deleted or supported by more clear data. What is the strong brown staining in normal brain? Which anatomical part of the brain is shown? It should be also stated what types of tumours are actually shown.*

As all referees have pointed out, our IHC pictures were with poor quality, which were taken using our own inverted microscope. We therefore re-captured all IHC images using an upright microscope and high resolution images are provided in this revised manuscript.

The cancer array we have used for Fig. 4A is a multiple organ tissue array, therefore the staining protocol may not optimal for each of the tissues contained on the same slide, though, optimizations were performed carefully. Higher background is seen with brain and skin samples, for example. However, the specific staining of the nucleus is evident and the enhanced staining of the tumour samples against normal tissues is also highly significant. The difference was much more impressive under the microscope in the same field.

The brown staining in normal brain is likely because of neuropile, which usually has non-specific staining. The cortex of brain is shown here. The types of tumours are indicated in revised Fig.4A.

We agree that the nuclear staining is more dominant and we eliminated the statement about cytoplasm staining.

Referee #2:

*The manuscript titled "SRSF1 and SRSF9 RNA binding proteins promote Wnt signalling-mediated tumourigenesis by enhancing β-catenin biosynthesis" by Yu Fu et al, describes important findings related to the regulation of beta catenin synthesis.*

Thank you for your support.

*1, The data in places requires further clarification. For example in Figure 3D the Axin2 control lane shows two bands but a single band in Srsf1-1 and Srsf1-2.*

Axin2 was only detected here as a target of Wnt signalling, we mistakenly considered the lower band as a non-specific one. When the previous results were re-evaluated, we realized that these two bands are both specific and both bands were down-regulated in two other experiments. The two

bands normally localized very close to each other that sometimes are seen as one band. In this particular experiment, the two were well separated probably because the gel was running too long. Since it is not absolutely required for this study, we decided to remove the Axin2 result. NCBI database search identifies no alternative splicing variant of human Axin2 gene and our preliminary result indicated that the upper band is not a phosphorylated form (data not shown). It is indeed an interesting question that why Axin2 has this two band pattern. Further investigation is needed.

*2, Further more the immunohistochemical data in figure 4A shows poorly the staining pattern of the antibodies as it is unclear in places where the staining is (cellular, nuclear or cytosoloic or extracellular). The colonic adenocarcinoma for examples doesn't show any gland formation.*

As all referees have pointed out, our IHC pictures were with poor quality, which were taken using our own inverted microscope. We therefore re-captured all IHC images using an upright microscope and high resolution images are provided in this revised manuscript.

The cancer array we have used for Fig. 4A is a multiple organ tissue array, therefore the staining protocol may not optimal for each of the tissues contained in the same slide, though, optimizations were performed carefully. Higher background is seen with brain and skin samples, for example. However, the specific staining of the nucleus is evident and the enhanced staining of the tumour samples against normal tissues is also highly significant. The difference was much more impressive under the microscope in the same field.

To have a full view of the sections, a lower magnification power was used for imaging.

*3, Also the introduction is not focused on the questions related to this manuscript and very general in places.*

Modified.

Referee #3:

*Fu and colleagues provide intriguing data that a subset of SRSF proteins regulate beta-catenin RNA stability and protein accumulation. The studies are well controlled and conducted in appropriate cell lines using both overexpression and knockdown systems.*

Thanks for your support.

*1, The authors show that cytoplasmic b-catenin is accumulated but not nuclear form after SRSF overexpression in Figure 1. This is an important point given that nuclear b-catenin mediates its oncogenic activity. This should be checked in colon cancer cell lines as well where the authors show SRSF inhibition of b-catenin pathway.*

Regarding SR over-expression-induced beta-catenin accumulation, we have a handful (or 4-5) repeats of each experiments and the results are splitted. Some showed a slight higher level of beta-catenin in the nuclear fraction, while the others not. In contrast, the enhanced cytosolic beta-catenin accumulation was constantly observed. In this situation, we have to conclude that, upon SR over-expression, only the cytosolic beta-catenin pool is significantly elevated.

Beta-catenin nuclear accumulation is a complex process under stringent regulation. It has been reported by several labs that Wnt stimulation not only triggers the stabilization and accumulation of beta-catenin protein, but also causes, via PI3K/Rac/JNK cascade, modifications of beta-catenin, e.g., phosphorylations, that enhances its nuclear translocation (Hu & Li, 2010; Tenbaum et al, 2012; Wu et al, 2008). These modifications could also be triggered, independent of Wnt ligand, by Ras/MAPK pathway as well, which are hyper-active in tumour cells (Guardavaccaro & Clevers, 2012; Janssen et al, 2006). In this regard, SR-induced beta-catenin should, at least in PI3K/Rac/JNK and Ras/MAPK low cell, stay mainly in the cytosol since it lacks the required modifications for nuclear transportation/retention. Off course this is not to say that these beta-catenin proteins will only stay in

the cytosol since their distribution could be influenced by the local environment such as growth factors. This is also consistent with the observation that in HCT116 cells, enhanced beta-catenin accumulation was observed in both fractions (Referee Figure 4), since in these cancer cells, the PI3K/Rac/JNK or RAS/MAPK pathways are hyper-active.

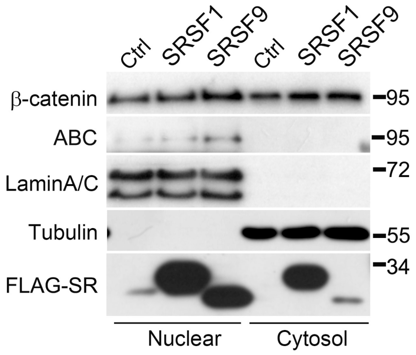


Referee Figure 4, fractionation of HCT116 cells over-expressing SR proteins.

To further clarify this issue, we detected the non-phosphorylated form, the so-called active form, of beta-catenin using the ABC antibody in these cell fractions. It seems that this active form of beta-catenin is slightly but constantly higher in the nuclear fraction after SR over-expression (Fig. 1D, 5A, 5B, Referee Figure 4). These results suggest that SR over-expression promotes cytocolic accumulation of beta-catenin and this may further leads to elevation of the transcriptional active form of beta-catenin in the cell nucleus.

Moreover, it is reported recently by Piccolo and colleagues (Azzolin et al, 2012) that beta-catenin has a cytosolic function in mediating stability control of TAZ, which is also tumorigenic. Although, in that paper it was not shown whether elevation of beta-catenin level could enhance TAZ accumulation, according to their working model, this is likely to happen. When beta-catenin level reaches the threshold exceeding Trcp-mediated degradation, it will likely protect TAZ from degradation. In this case, TAZ may mediate some of the cell transformation activity of cytosolic accumulated beta-catenin.

Finally, we have to accept that SR-induced beta-catenin accumulation is much less than that from Wnt stimulation or APC mutation. Therefore, we believe that beta-catenin is just one of the key target proteins involved in SR-over-expression-induced cell transformation. Our loss-of-function results (Fig. 5C) also indicated that knockdown beta-catenin just partially reduced cell transformation efficacy by SR proteins. These targets, including BIN1, MNK2, survivin, beta-catenin and other unidentified ones, synergistically promote tumourigenesis in SR-over-expression cells.

*2, Authors suggest that SRSF1 and SRSF9 promote beta-catenin accumulation in a mTOR dependent mechanism (Figure 2E). The p-S6K kinase activity does not correlate too well as SRSF9 shows more S6K phospohorylation (Figure 2E) but less robust beta-catenin accumulation (Fig. 1C). A complete analysis of SRSF proteins (1-12) would be informative in drawing a more significant conclusion as to whether the two groups of SRSF proteins (1,3,5,7,8,9,10,12 vs 2,4,6,11) show similar differences in mTOR pathway activity. The authors' conclusion on page 11, line 5 is overstated. It is not clear from this data that these effects are "mTOR-dependent". To make this conclusion more decisive, further data should be provide - e.g. using mTOR inhibitors in the presence/absence of SRSF1/9 to determine whether b-catenin accumulation is blocked.*

Regarding promoting beta-catenin accumulation, and S6K phosphorylation, SRSF1 and SRSF9 have in fact similar activities. One problem of SRSF9 is that its production upon transfection in many cases is lower than SRSF1, for example, in Fig. 1C. However, when SRSF9 is really well produced, for example, in Fig. S2C, it is at least as active as SRSF1.

As suggested, we over-expressed all 12 SR proteins in HEK293T cells and analysed their activities on promoting S6K phosphorylation. This is a tough experiment but from the Referee Figure 5, we could roughly see that SRSF1, 2, 3, 5, 7, 8, 9 and10 are active ones and SRSF4, 6, 11 and 12 are inactive ones. Comparing with their beta-catenin promoting activities, SRSF2 and 12 showed

discrepancies while the rest were consistent. In these two experiments, the expression of SRSF12 was probably too low to trigger S6K activation. The SRSF2 is pretty active on S6K but inactive on beta-catenin. One explanation might be that this SR protein does not bind to beta-catenin mRNA, although, we cannot rule out other possibilities at this stage. This is consistent with the previous report that SRSF2 caused NIH3T3 cell transformation in colony formation assays but failed in xenograft assays(Karni et al, 2007). It may, through mTOR activation, promote other targets that lead to cell transformation. In general, these preliminary results support the conclusion that SR's activities on promoting beta-catenin accumulation and S6K activation are largely correlated. We are currently testify all SR proteins regarding their cell transformation activities and hope to get a comprehensive view about tumourigenesis relations of all SR protein family members.

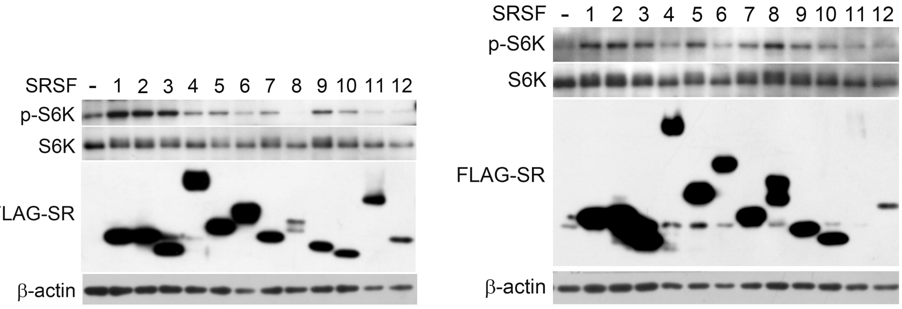


Referee Figure 5, S6K activation after SR over-expression in HEK293T cells.

As suggested, we applied mTOR inhibitors Rapamycin to inhibit SR-promoted beta-catenin accumulation in transfected HEK293T cells. As shown in Referee Figure 6, SRSF1 or SRSF9 enhanced beta-catenin production was indeed reduced, however, beta-catenin production itself without over-expressed SR was also inhibited. So it's impossible to draw a conclusion.

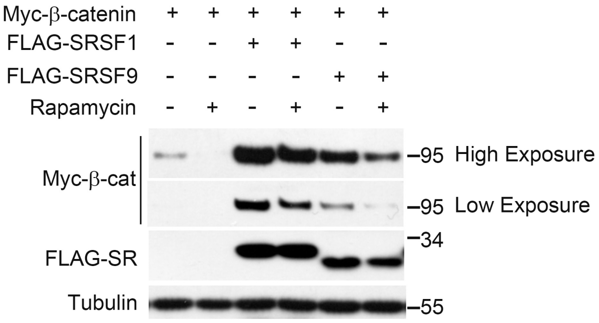


Referee Figure 6, Rapamycin blocks beta-catenin production.

Our conclusion that SR promoted beta-catenin accumulation is "mTOR-dependent" is based on the following evidences:

1) We have been working very hard and for a long time (over one year) to rule out the possibility that SR affected beta-catenin stability. This is so obvious once you get more beta-catenin after a treatment, however, all the results we obtained did not suggest that it was the stability that was changed upon SR co-expression. We think these results also largely excluded many other indirect possibilities, for example, via alteration of Axin or other major components of the beta-catenin destruction complex. The conclusion that beta-catenin's stability/degradation was not changed by SR was convincingly demonstrated that the half life of beta-catenin was not changed upon SR over-expression (Fig. 2A and S2A) and that the stabilized beta-catenin was further elevated by SR proteins (Fig. S2C).
2) We have also excluded the possibility that SR over-expression affected beta-catenin mRNA (Fig. S2B).
3) SRSF1 over-expression is able to activate mTOR and the mechanism was well studied (Karni et al, 2008; Michlewski et al, 2008). We just show that SRSF9 is also able to do so (Fig. 2E).
4) Domain analysis indicated that, for beta-catenin promotion, the nuclear localization of SR protein and the splicing activity (the RS domain is necessary for both) were not essential, yet, the AAA mutant lacking mTOR activating capacity was completely dead. As shown in Referee Figure 7, the full length SRSF9 is largely a nuclear protein and so is the AAA mutant. In

contrast, the mutants without RS domain are much more cytosolic. Domain analysis provided strong evidences that the mTOR activation is critical for SR proteins' action on beta-catenin.

5) SRSF1 and SRSF9 proteins are able to bind beta-catenin mRNA and to enhance incorporation of beta-catenin mRNA into ribosome fractions.

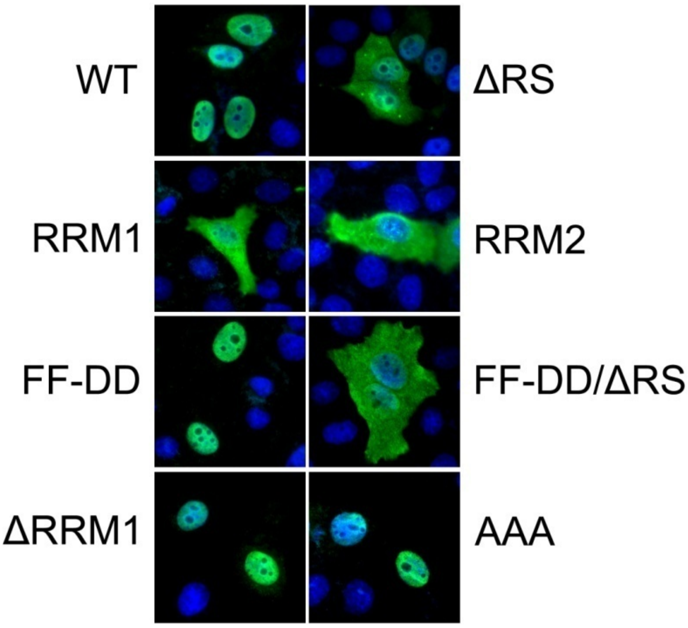


Referee Figure 7, cellular distribution of SRSF9 mutants in transfected HeLa cells. Green signals indicated SR proteins and blue indicated DNA.

*4, Authors show intriguing data that colon cancer cell lines reduce b-catenin levels after SRSF1/9 siRNA inhibition. Published data shows that b-catenin active cell lines such as HCT-116 and SW480 are dependent on b-catenin activity for proliferation. Since the ability of SRSF RNAi to reduce b-catenin protein appears mild (Figure 3D-E), it is important to know whether effects on cell proliferation were seen in these models. It is not clear why soft agar colony assays were used as a read-out in Figure 6.*

We used soft agar colony formation assay to show that SRSF1 and SRSF9 are required for tumorigenic activity of these colon cancer cells. We believe colony formation capacity is a more global view of tumourigenesis, which is the net outcome of proliferation, survival, cell-cell contact and so on. Nevertheless, we agree that MTT assay is a more direct evaluation of cell proliferation and we performed these experiments with HCT116 cells after transfection of siRNAs against SRSF1 or SRSF9. The results were included in revised Fig. 6A-B. Knockdown SRSF1 or SRSF9 indeed reduced cell proliferation, accompanied with down-regulation of beta-catenin.

As the response to Referee #1 Question 2, we think the level of contribution of different SR proteins on beta-catenin synthesis is slightly different in various cell lines. beta-catenin down-regulation in RKO and HCT116 after SRSF1 siRNA is pretty significant (Fig. 3B, 3D and 6C), while that after SRSF9 was relatively marginal (Fig. 3C, 3E and 6D).

*5, The immunohistochemical staining shows that this antibody is non-specific and displays background signal. For example, strong keratin staining is seen in the epidermis. The fact that in some tissues cytoplasmic staining is seen but in other nuclear may reflect non-specific staining rather than target distribution changes as the authors suggest. The authors should provide validation and supporting evidence that this antibody is specifically detecting SRSF9 in tissues. For example, do RNA based studies (microarray or qPCR based) confirm SRSF9 overexpression in the tumour type indicated? Does the IHC signal disappear in cell line pellets after SRSF9 knockdown ?*

As all referees have pointed out, our IHC pictures were with poor quality, which were taken using our own inverted microscope. We therefore re-captured all IHC images using an upright microscope and high resolution images are provided in this revised manuscript.

The cancer array we have used for Fig. 4A is a multiple organ tissue array, therefore the staining protocol may not optimal for each of the tissues contained in the same slide, though, optimizations were performed carefully. Higher background is seen with brain and skin samples for example. However, the specific staining of the nucleus is evident and the enhanced staining of the tumour samples against normal tissues is also highly significant. The difference was much more impressive under the microscope in the same field.

The brown staining in normal brain is likely because of neuropile, which usually has non-specific staining. The types of tumours are indicated in revised Fig. 4A.

We agree that the nuclear staining is more dominant and we eliminated the statement about cytoplasm staining.

Regarding the specificity of the SRSF9 antibody, we believe it is specific enough. The IHC experiments were performed by a service team which is very experienced on IHC and the results were analysed by an independent pathologist. This multiple organ tissue array contained normal and tumour samples from 20 different tissues and 16 of them did not shown specific elevation of SRSF9 expression, which we did not show in our manuscript. Only the four types that showed specific over-expression were mentioned. To further verify the specificity of this antibody, we performed IHC on cover slide containing SW620 and SW620-Srsf9-shRNA stable line cells. As shown in Referee Figure 8, the staining on knockdown cells is clearly weaker.

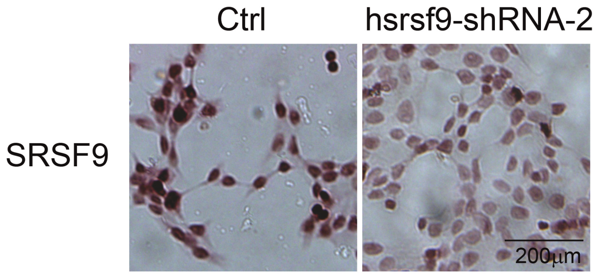


Referee Figure 8, IHC with SRSF9 antibody.

To further confirm that Srsf9 gene is indeed over-expressed in tumour samples, we searched the Oncomine cancer microarray database (http://www.oncomine.com), and found a significant increase ($P<0.05$) of SRSF9 (SRp30c) expression in tumour tissues over the expression in corresponding normal tissues in 156 of 360 studies. Fisher's meta analysis indicated that the observed increase in those paired studies was extremely significant (meta p-value<0.0001). This result suggested that Srsf9 gene is over-expressed in certain tumour samples and the up-regulation happens at its mRNA level.

*6, Page 14, 2nd paragraph: the correlation between b-catenin overexpression and SRSF overexpression does not appear to be statistically significant as the same percent of tumours were b-catenin high regardless of SRSF1 expression. The authors present data showing that many SRSF proteins can mediate b-catenin accumulation so does it make sense that only one SRSF protein will correlate to its expression in colon cancer ? A more robust bio-informatic analysis of SRSF protein expression and b-catenin expression would be required beyond what is presented here.*

You are absolutely right that the correlation between SRSF1 over-expression and beta-catenin accumulation is not statistically significant (fisher's test, $P=0.22$). Moreover, it is obvious that SR protein over-expression is NOT the major driving force that causes beta-catenin accumulation in colon cancer samples. APC mutations are the major effectors and beta-catenin mutations contribute to less extend. However, that is not to say that SR proteins do nothing. As we mentioned in Discussion, SR overexpression-mediated higher beta-catenin synthesis, as a background, may enhance beta-catenin accumulation upon APC or beta-catenin mutation. We are currently testing this hypothesis and hope to further clarify the interaction between SR-mediated elevation of beta-catenin synthesis and mutant APC-mediated degradation defects.

© EMBO

Ideally, the tumour samples should be firstly separated according to their APC and beta-catenin status, e.g., wild type or mutant, and then the correlation between SR over-expression and beta-catenin ectopic accumulation be testified. This strategy would imply genomic analysis and then mRNA and protein verifications. It is practically very difficult to perform in large scale.

Since this result is just supportive, other than decisive, to our main conclusion, we decided to remove this result.

*Minor points:*
*Page 12, 2nd paragraph, the data focuses on SRSF9 but authors write "....and SRSF1 has been suggested....)*

The results were about SRSF9 but we started from mentioning parallel results published by other groups just to inform the readers that, regarding SRSF1, this was known.

*Authors interchange SRSF and SRSP (page 12, second to last line).*

Corrected.

*Figure 4B: the SRSF1 blot is massively over-exposed and impossible to determine the degree of exogenous SRSF1 overexpression compared to endogenous levels.*

Corrected.

*Figure 5F : the resolution of the images provided is low. Higher resolution photos should be provided.*

Panel removed.

Reference

Azzolin L, Zanconato F, Bresolin S, Forcato M, Basso G, Bicciato S, Cordenonsi M, Piccolo S (2012) Role of TAZ as mediator of Wnt signaling. Cell 151: 1443-1456

Guardavaccaro D, Clevers H (2012) Wnt/beta-catenin and MAPK signaling: allies and enemies in different battlefields. Sci Signal 5: pe15

Hu T, Li C (2010) Convergence between Wnt-beta-catenin and EGFR signaling in cancer. Molecular cancer 9: 236

Janssen KP, Alberici P, Fsihi H, Gaspar C, Breukel C, Franken P, Rosty C, Abal M, El Marjou F, Smits R et al (2006) APC and oncogenic KRAS are synergistic in enhancing Wnt signaling in intestinal tumor formation and progression. Gastroenterology 131: 1096-1109

Karni R, de Stanchina E, Lowe SW, Sinha R, Mu D, Krainer AR (2007) The gene encoding the splicing factor SF2/ASF is a proto-oncogene. Nature structural & molecular biology 14: 185-193

Karni R, Hippo Y, Lowe SW, Krainer AR (2008) The splicing-factor oncoprotein SF2/ASF activates mTORC1. Proc Natl Acad Sci U S A 105: 15323-15327

Michlewski G, Sanford JR, Caceres JF (2008) The splicing factor SF2/ASF regulates translation initiation by enhancing phosphorylation of 4E-BP1. Molecular cell 30: 179-189

Tenbaum SP, Ordonez-Moran P, Puig I, Chicote I, Arques O, Landolfi S, Fernandez Y, Herance JR, Gispert JD, Mendizabal L et al (2012) beta-catenin confers resistance to PI3K and AKT inhibitors and subverts FOXO3a to promote metastasis in colon cancer. Nat Med 18: 892-901

Wu X, Tu X, Joeng KS, Hilton MJ, Williams DA, Long F (2008) Rac1 activation controls nuclear localization of beta-catenin during canonical Wnt signaling. Cell 133: 340-353

Accepted 08 March 2013

Please find enclosed the final reports on your manuscript. We are pleased to inform you that your manuscript is accepted for publication and is being sent to our publisher to be included in the next available issue of EMBO Molecular Medicine.

Congratulations on your interesting work,

***** Reviewer's comments *****

Referee #1:

The manuscript is now suitable for publication.

Referee #3:

The authors have addressed the reviewers' comments to my satisfaction and publication is warranted.
