# Supplementary material for: SRSF1 and SRSF9 RNA binding proteins promote Wnt signalling-mediated tumorigenesis by enhancing β-catenin biosynthesis
Source: EMBO Mol Med. 2013 Apr 17;5(5):737–50. doi: 10.1002/emmm.201202218 (PMC3662316; doi:10.1002/emmm.201202218)
Supplement: Supplementary file 2 [file emmm0005-0737-sd2.pdf]

Supplemental Sections:

Three Supplemental Figures and Figure legends.

Supplemental Figure 1. SRSF1 and SRSF9 specifically promote Wnt/β-catenin signaling.

Supplemental Figure 2. Mechanism of β-catenin accumulation induced by SR proteins.

Supplemental Figure 3. Expression of SR proteins in human colon cancer cell lines.

Fu et al. 2012 Figure S1

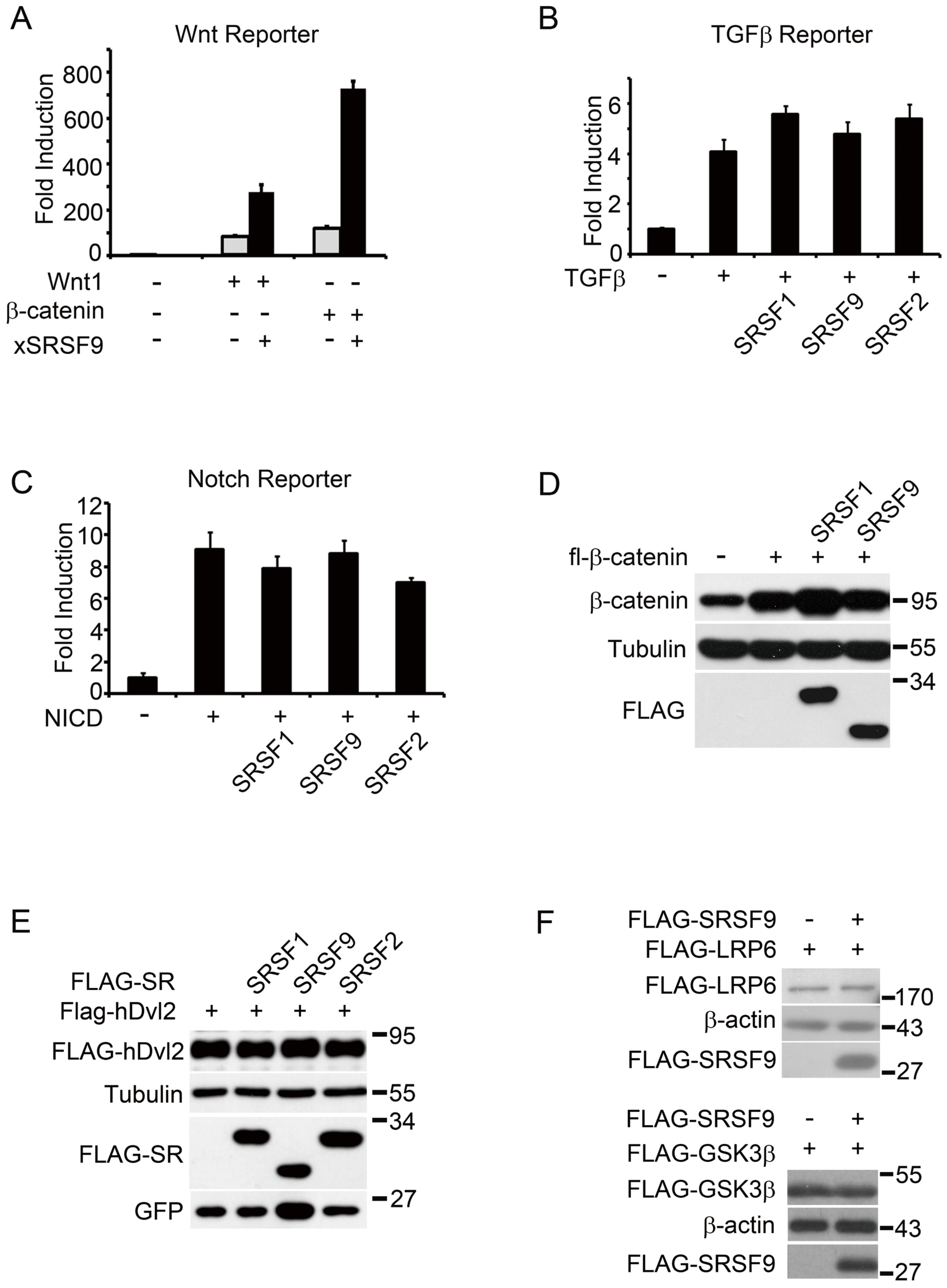

Figure S1. SRSF1 and SRSF9 specifically promote Wnt/β-catenin signaling.

(A) *Xenopus* SRSF9 (xSRSF9) was able to enhance Wnt1- and β-catenin-activated signaling. HEK293T cells were transfected with TOPFLASH luciferase reporter, xSRSF9, Wnt1 or β-catenin as indicated.

(B) SR proteins did not enhance TGFβ reporter expression. HEK293T cells were transfected with CACG luciferase reporter, SR plasimids as indicated and cells were subsequently stimulated with TGFβ protein for 12h and then luciferase reporter activity was measured.

(C) SR proteins did not enhance Notch reporter expression. HEK293T cells were transfected with pGa981-6 luciferase reporter, SR plasmids and NICD (Notch intracellular domain, which activates Notch signaling) as indicated and luciferase activities were measured 36h later.

(D) SRSF1 and SRSF9 were able to elevate β-catenin protein production from a full length human β-catenin cDNA (fl-β-catenin). The β-catenin specific antibody was used and both endogenous as well as exogenous proteins were detected at the same migration position.

(E) The protein level of FLAG-Dvl2 was not affected by over-expression of SR proteins. Tubulin was shown as loading control.

(F) The protein level of FLAG-LRP6 and FLAG-GSK3β were not affected by over-expression of SRSF9. β-actin was used as loading control.

Fu et al. 2012 Figure S2

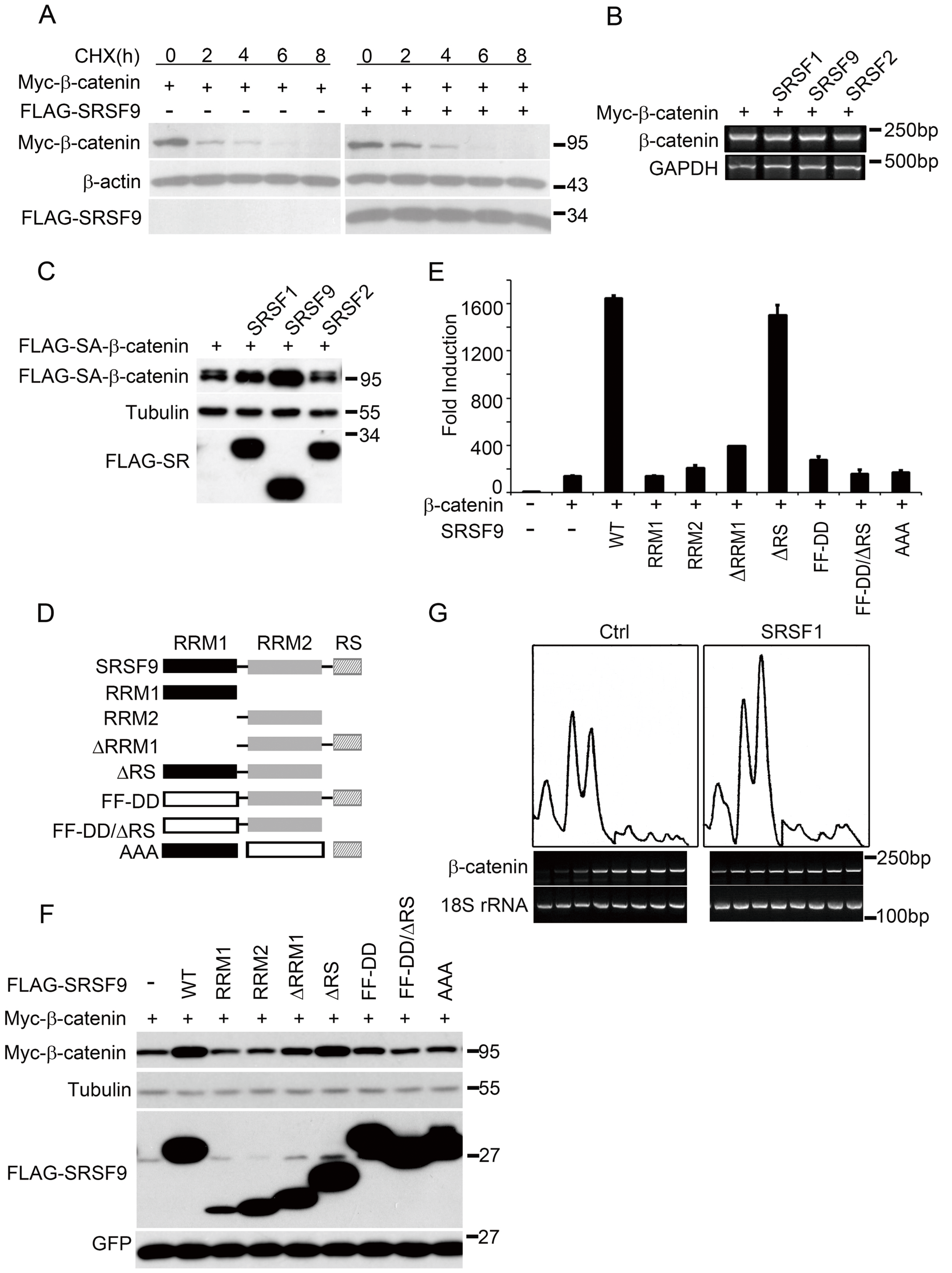

Figure S2. Mechanism of β-catenin accumulation induced by SR proteins

(A) β-catenin protein stability was not affected by over-expressed SRSF9. Myc-β-catenin was transfected alone or co-transfected with FLAG-SRSF9 and 36h later cells were treated with cycloheximide (CHX, 100μg/mL) and then harvested at the indicated time points (hours post adding). In order to have the same β-catenin level at the starting point, 300ng β-catenin plasmid was used for tranfection alone and 200ng for co-transfection with 10ng SRSF9 plasmid.

(B) Over-expression of SR proteins did not affect β-catenin mRNA level. HEK293T cells were transfected as indicated and 36h later, total RNA was extracted and RT-PCR was performed to detect β-catenin mRNA level. GAPDH was used as a loading control.

(C) The protein level of SA-β-catenin (a mutant form resistant to degradation system) was elevated by over-expressed SRSF1 or SRSF9, but not SRSF2. Tubulin was used as a loading control.

(D) Schematic diagram showing SRSF9 deletions and mutations.

(E) TOPFLASH reporter assay results showing that SRSF9 and its deletions/mutations exerted different activities on β-catenin signaling. WT means wide type.

(F) SRSF9 and its deletions/mutations exerted different activities on β-catenin protein production. Myc-β-catenin/GFP mix was transfected alone or co-transfected into HEK293T cells with indicated SRSF9 mutations/deletions and the total cell lysates were proceeded by SDS-PAGE and Western blotting. WT means wide type.

(G) Overexpression of SRSF1 enhances translation of β-catenin mRNA. Cell lysates from HEK293T cells transfected with SRSF1 or empty vector (Ctrl) were fractionated across a 10%-45% sucrose gradient. The RNA was extracted from the fractions and RT-PCR was performed to detect β-catenin mRNA. (Top) UV absorbance (254nm) profile of cytosolic ribonucleoprotein complexes.

Fu et al. 2012 Figure S3

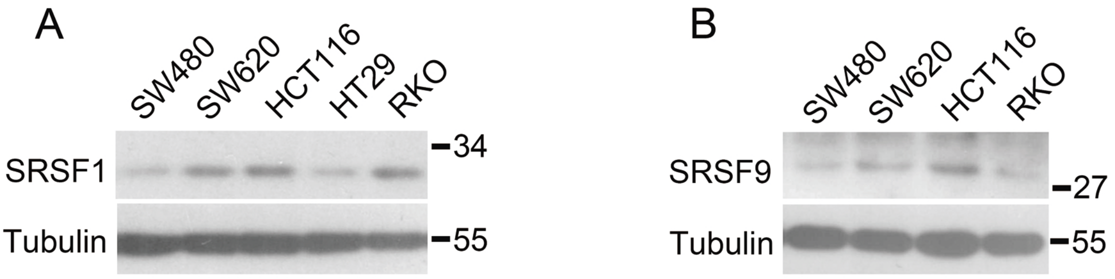

Figure S3. Expression of SR proteins in human colon cancer cell lines

(A) Expression of SRSF1 in human colon cancer cell lines. Tubulin was used as a loading control.

(B) Expression of SRSF9 in human colon cancer cell lines. Tubulin was used as a loading control.
